# Machine learning-based spatial characterization of tumor-immune microenvironment in the EORTC 10994/BIG 1-00 early breast cancer trial

**DOI:** 10.1038/s41523-025-00730-1

**Published:** 2025-03-07

**Authors:** Ioannis Zerdes, Alexios Matikas, Artur Mezheyeuski, Georgios Manikis, Balazs Acs, Hemming Johansson, Ceren Boyaci, Caroline Boman, Coralie Poncet, Michail Ignatiadis, Yalai Bai, David L. Rimm, David Cameron, Hervé Bonnefoi, Jonas Bergh, Gaetan MacGrogan, Theodoros Foukakis

**Affiliations:** 1 Department of Oncology-Pathology, Karolinska Institutet, Stockholm, Sweden; 2 Theme Cancer, Karolinska Comprehensive Cancer Center and University Hospital, Stockholm, Sweden; 3 Breast Center, Theme Cancer, Karolinska Comprehensive Cancer Center and University Hospital, Stockholm, Sweden; 4 Department of Immunology, Genetics, and Pathology, Uppsala University, Uppsala, Sweden; 5 Molecular Oncology Group, Vall d’Hebron Institute of Oncology, Barcelona, Spain; 6 Computational BioMedicine Laboratory (CBML), Foundation for Research and Technology-Hellas (FORTH), Heraklion, Greece; 7 Department of Clinical Pathology and Cancer Diagnostics, Karolinska University Hospital, Stockholm, Sweden; 8 European Organisation for Research and Treatment of Cancer Headquarters, Brussels, Belgium; 9 Department of Medical Oncology, Institut Jules Bordet and L’Université Libre de Bruxelles (U.L.B), Brussels; Academic Trials Promoting Team (ATPT), Institut Jules Bordet, Brussels, Belgium; 10 Department of Pathology, Yale School of Medicine, New Haven, CT USA; 11 Yale Cancer Center, Yale School of Medicine, New Haven, CT USA; 12 Edinburgh University Cancer Centre, Institute of Genetics and Cancer, University of Edinburgh, Edinburgh, UK; 13 Department of Medical Oncology, Institut Bergonié Unicancer, INSERM U1312, Université de Bordeaux, Bordeaux, France; 14 Department of Biopathology, Institut Bergonié Unicancer, INSERM U1312, Bordeaux, France

**Keywords:** Breast cancer, Cancer microenvironment

## Abstract

Breast cancer (BC) represents a heterogeneous ecosystem and elucidation of tumor microenvironment components remains essential. Our study aimed to depict the composition and prognostic correlates of immune infiltrate in early BC, at a multiplex and spatial resolution. Pretreatment tumor biopsies from patients enrolled in the EORTC 10994/BIG 1-00 randomized phase III neoadjuvant trial (NCT00017095) were used; the CNN11 classifier for H&E-based digital TILs (dTILs) quantification and multiplex immunofluorescence were applied, coupled with machine learning (ML)-based spatial features. dTILs were higher in the triple-negative (TN) subtype, and associated with pathological complete response (pCR) in the whole cohort. Total CD4+ and intra-tumoral CD8+ T-cells expression was associated with pCR. Higher immune-tumor cell colocalization was observed in TN tumors of patients achieving pCR. Immune cell subsets were enriched in *TP53*-mutated tumors. Our results indicate the feasibility of ML-based algorithms for immune infiltrate characterization and the prognostic implications of its abundance and tumor-host interactions.

## Introduction

Breast cancer (BC) represents a clinically and biologically heterogeneous disease ecosystem. Further characterization of the tumor microenvironment (TME), its components and molecular drivers could elucidate the complexity of tumor-host interactions and provide rationale for biomarker development. One of the principal TME components, tumor-infiltrating lymphocytes (TILs), are both prognostic for outcomes and predictive for response to chemotherapy^1–5^.

Our understanding of the immune cell composition and spatial interactions within the TME is evolving. Towards this end, artificial-intelligence (AI) and machine-learning (ML) methods—which have revolutionized digital pathology and diagnostics—could represent valuable tools for studying the TME^6,7^. Indeed, recent studies have reported on the performance, prognostic implications, advantages, and challenges of digital-assisted scoring of TILs and multiplex immunofluorescence methods for TME characterization in BC^8,9^. However, the spatial relationships between the various components of immune infiltrate and tumor cells still warrant further investigation.

The aims of this study were i) to comprehensively and objectively characterize the composition of tumor-immune microenvironment landscape at a spatial level, ii) explore its distribution according to *TP53* mutational status and iii) evaluate relevant prognostic implications by using automated, ML-based and multispectral spatial imaging approaches in the context of a large randomized phase III trial at the neoadjuvant setting^10^.

## Results

### Patient characteristics

**Fig. 1:**
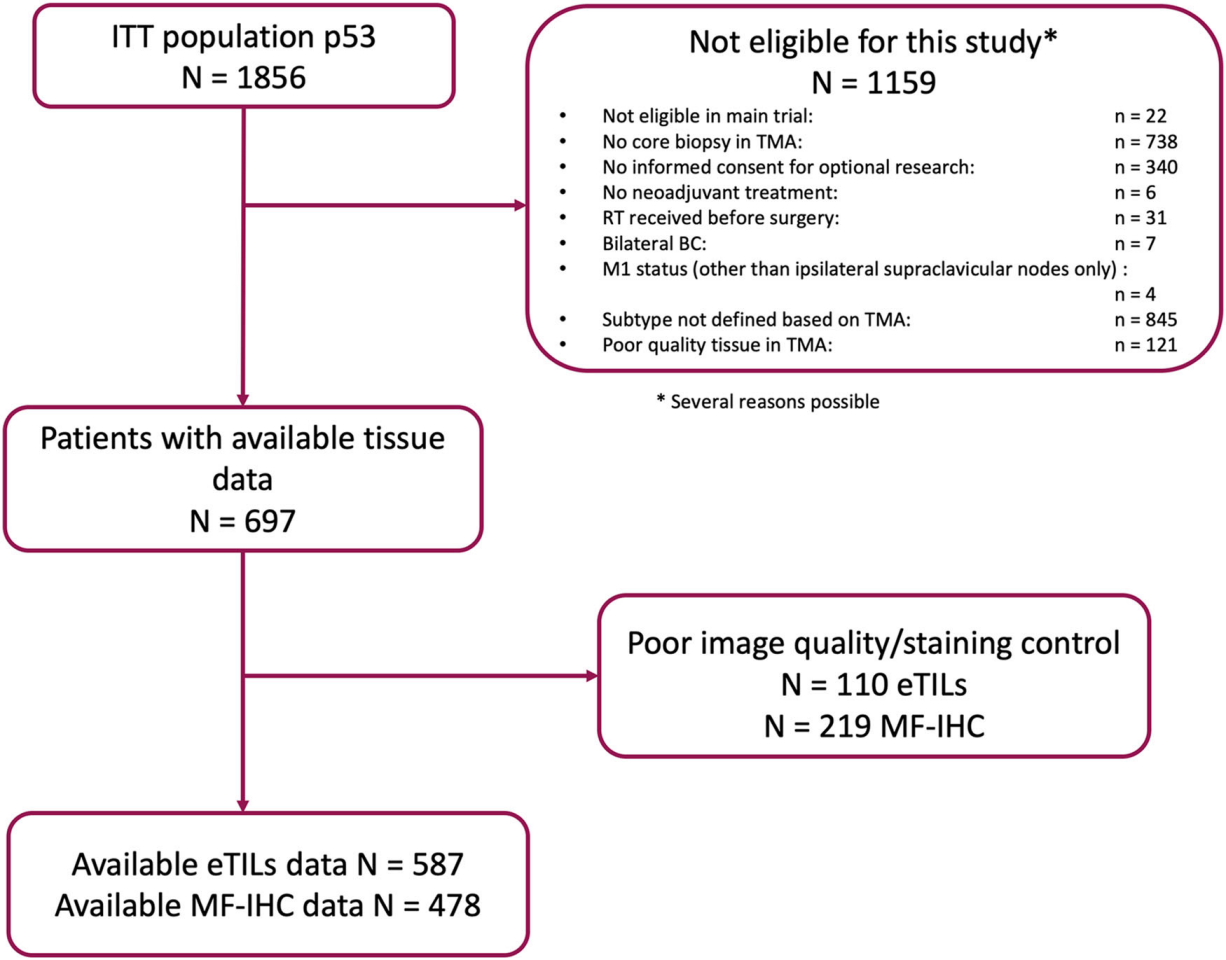
Patient disposition and data availability of the study. Flowchart depicting the data availability for the translational analyses in the present study; ITT intention-to-treat, eTILs digitally-assessed tumor-infiltrated lymphocytes, MF-IHC multiplex fluorescent immunohistochemistry, TMA tissue microarrays.

Out of the 1856 patients initially enrolled in the trial, 697 had available tissue and were eligible for this study. Upon staining and tissue quality assessment, 587 and 478 patients had available digital TIL (dTIL) and multiplex immunofluorescence data, respectively and included in the final analysis. The patient disposition presenting the eligibility criteria and data availability is shown in Fig. 1. The demographic/clinicopathologic characteristics of the different patient populations according to the methods used are presented in Supplementary Table 1. At the time of the latest data cutoff (9th October 2018), median follow-up was 11.4 years (interquartile range = 10.16–12.41 years).

### Digital image analysis-based enumeration of TILs and correlation with outcomes

**Fig. 2:**
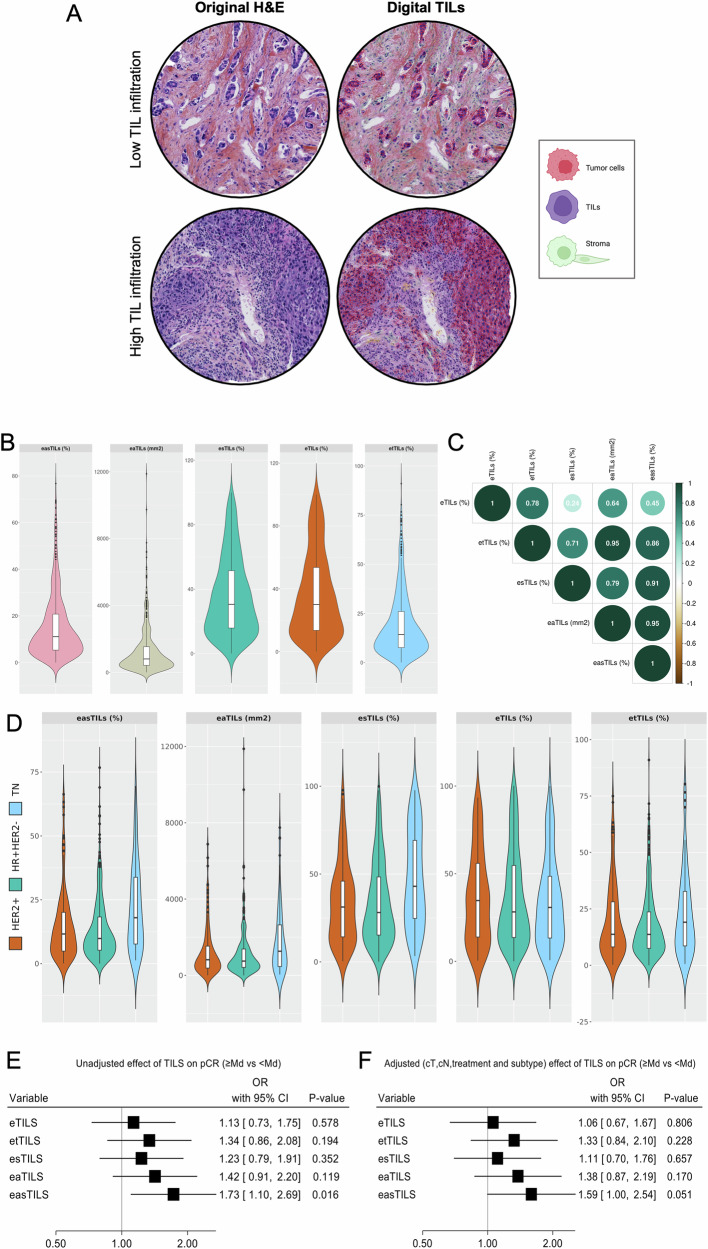
Digital TILs evaluation using a machine-learning algorithm in archival H&E-stained FFPE breast cancer tissue microarrays. **A** Representative TMA images of digital TILs enumeration in patients with low and high immune infiltration, created with BioRender.com; **B** Distribution of the digital TILs variables in the whole population (*n* = 587); **C** Correlation matrix for the different dTILs variables, Spearman’s rank correlation coefficient; **D** Distribution of dTILs variables within IHC-based subtypes; Forest plots on prognostic effect of dTILs on pCR in the univariate (**E**) and multivariable (**F**) logistic regression model (adjusted for tumor size, nodal status, treatment and stratified by subtype) in the whole cohort.

Using the previously validated CNN11 image-analysis algorithm on hematoxylin & eosin (H&E)-stained tissue microarrays (TMA), we calculated different digital TILs (dTILs) metrics based on the various cell annotations (Fig. 2A). Among the dTILs variables, the median expression of easTILs was 11% (range: 0.01–76.7) (Fig. 2B, Supplementary Table 2). The different dTILs metrics were overall strongly and statistically significantly correlated with each other (Fig. 2C). DTILs abundance was generally higher in the triple-negative subtype compared to the HER2+ and HR+/HER2- (Fig. 2D, Supplementary Table 2).

Regarding associations with patient outcomes, easTILs were associated with pCR rates both in univariate (OR_unadjusted _= 1.73, 95% CI 1.10–2.69, *p* = 0.016) and multivariable (OR_adjusted_= 1.59, 95% CI 1.00–2.54, *p* = 0.05) analysis (Fig. 2E, F). There was a significant interaction between easTIL and IHC-based subtypes for pCR (*p* = 0.045), with the association mainly observed in the HR+/HER2- subtype. However, no significant association with PFS was noted (Supplementary Fig. 1).

### Immune cell landscape composition at a multiplex resolution and correlation with outcomes

**Fig. 3:**
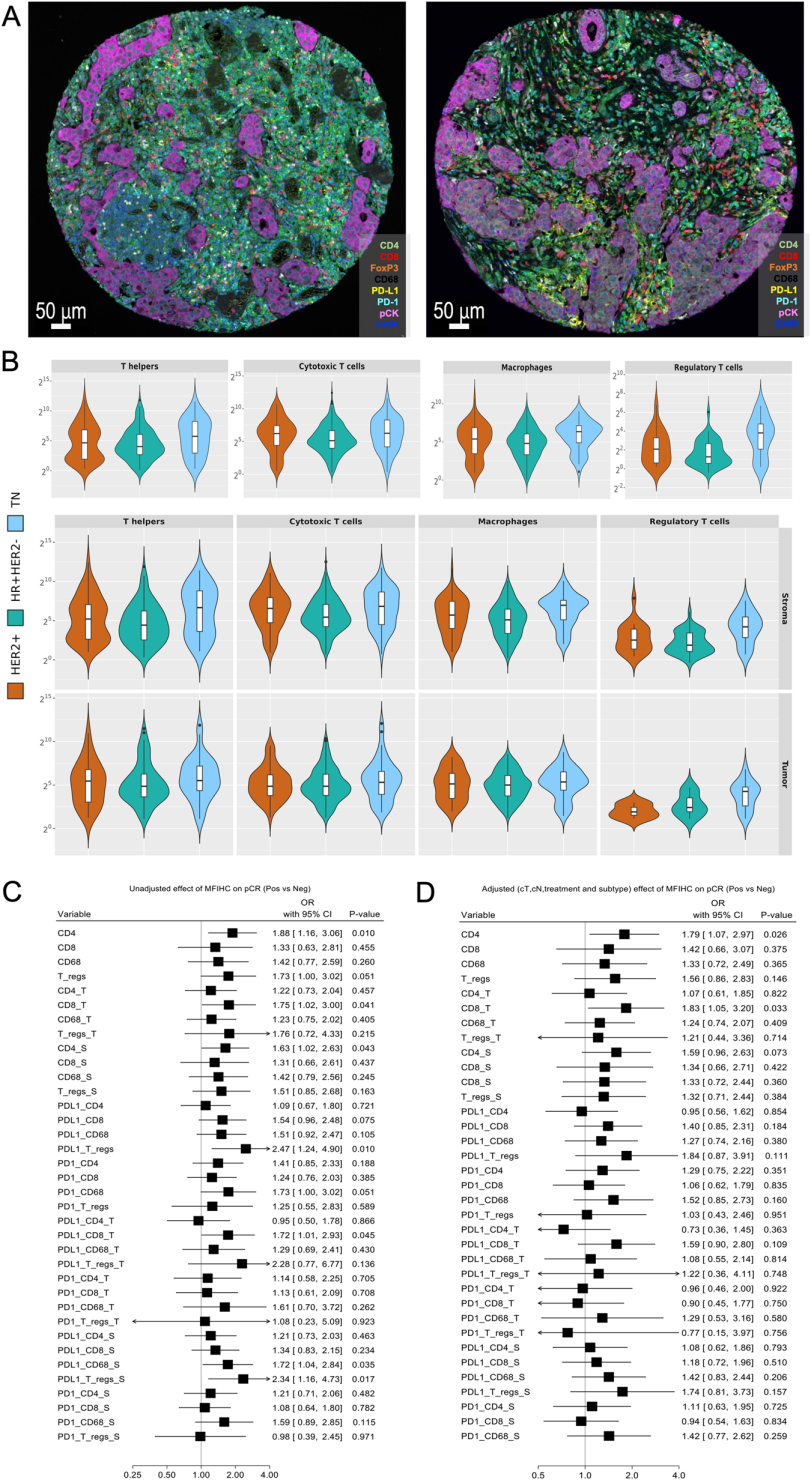
Characterization of the immune infiltrate at a multiplex resolution. **A** Representative stained images with the multiplex immunofluorescence antibody panel; **B** Distribution of cell densities/immune cell subsets for total area (upper panel), stroma (middle panel) and tumor (lower panel) compartments within IHC-based subtypes. All values are log2-transformed; Forest plots on prognostic effect of multiplex immunofluorescence immune cell subpopulations on pCR in the univariate (**C**) and multivariable (**D**) logistic regression model (adjusted for tumor size, nodal status, treatment and stratified by subtype) in the whole cohort.

To further characterize the specific composition of the immune infiltrate, we performed multiplex immunofluorescence in the available TMA using an immune-related antibody panel. We evaluated the abundance (i.e., defined as normalized cell densities = number of marker positive cells/mm^2^) and localization (total, tumor and stroma area) of the different immune cell subpopulations based on i) the expression of single markers (CD4+ for T-helpers, CD8+ for cytotoxic T-cells, FoxP3+ for T-regulatory cells, CD68+ for macrophages) and ii) the respective co-expression of PD-L1 and PD-1 checkpoint markers on these cells (Fig. 3A, Supplementary Table 3). Upon the application of an established workflow and tissue/staining quality control, immune cell quantification could be performed in a total of 478 patients (*n* = 468 for tumor area analysis; 10 patients had only stroma area for analysis).

In the whole patient population, CD8+ cytotoxic T-cells were the most abundant cell subset in the total, intra-tumoral and stromal areas. Both PD-1 and PD-L1 checkpoint expression was highest in CD4 + T-cells (T-helpers), followed by cytotoxic T-cells (CD8+) in all tissue compartments (Supplementary Table 3). Within IHC-based subtypes, the mean cell densities of all immune cell subpopulations (i.e., T-cell and macrophages) and also the respective co-expression of the PD-1/PD-L1 markers were significantly enriched in the triple-negative compared to the other subtypes, regardless of tissue localization (Supplementary Table 4, Fig. 3B). Furthermore, the different immune cell subsets derived from the multiplex immunofluorescence analysis were correlated both with each other and with digital TILs (Supplementary Fig. 2).

Among the different cell subpopulations, CD4 + T-helpers in the total area (ORadj = 1.79, 95% CI 1.07–2.97, *p* = 0.026) and intra-tumoral CD8+ cytotoxic T-cells (ORadj = 1.83, 95% CI 1.05–3.20, *p* = 0.033) were associated with improved pCR (Fig. 3C, D). No significant association with PFS was observed for any marker (Supplementary Fig. 3).

### Spatial composition of immune infiltrate, cell-to-cell interactions, and correlation with pathologic complete response in the triple-negative subtype

**Fig. 4:**
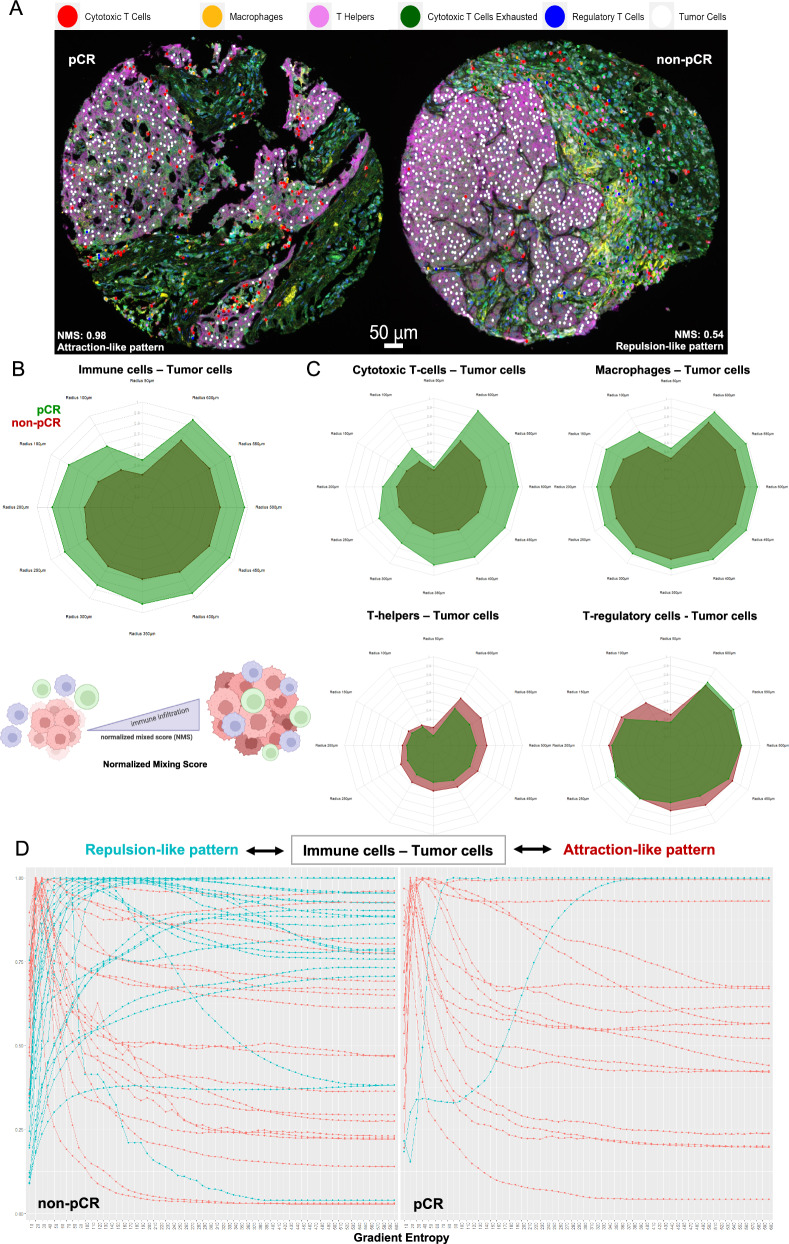
Spatial characterization of the tumor-immune cell interactions (using ML-based metrics) in the triple-negative subtype in patients that achieved pCR versus non-pCR. **A** Representative images depicting the spatial phenotyping and interactions among different immune cell subpopulations with tumor cells in a pCR (left) and a non-pCR (right) patient; Radar plots depicting normalized mixing score (NMS) values for different increasing radii for the interaction of overall immune-tumor cells (**B**) and the specific immune cell subpopulations in patients achieving pCR (green) versus non-pCR (red) TN (**C**). **D** Entropy gradient slope depicting attraction-like (red lines) and repulsion-like (blue lines) patterns for the interaction between immune and tumor cells in non-pCR (left panel) versus pCR (right panel) patients. Figure 3B was created with *BioRender.com*.

To further explore the spatial distribution and complexity of immune-tumor host interactions, we calculated two different ML-based metrics using the multiplex immunofluorescence cell data, namely i) the normalized mixing score (NMS) and ii) the entropy gradient. Given that the abundance of immune cells was highest in the triple-negative subtype, we focused on the description of their spatial patterns in this patient subgroup. Higher baseline NMS values for the immune-tumor cell interactions were observed in patients that achieved pCR compared to the non-pCR ones, regardless of radius (Fig. 4A, B), indicating higher degree of cell–cell colocalization for the pCR patients. This effect was maintained when we tested separately the interactions of tumor cells with cytotoxic T-cells or macrophages, but not for T-helpers or regulatory T-cells (Fig. 4C, Supplementary Table 5). Similarly, an “attraction-like” entropy gradient slope pattern for the interaction of immune with tumor cells was observed mostly in patients that achieved pCR compared to the non-pCR patients who had an enriched “repulsion-like pattern” (Chi-squared *p* = 0.01; Fig. 4D). This effect was observed for the interactions of tumor cells with most of the immune cell subpopulations, except for macrophages (Supplementary Fig. 4). Taken together, by using two different ML-based approaches we observed that closer spatial interaction between tumor and immune cells correlated with higher probability of pCR in TNBC subtype.

### Association of *TP53* mutational status with immune infiltrate components

**Fig. 5:**
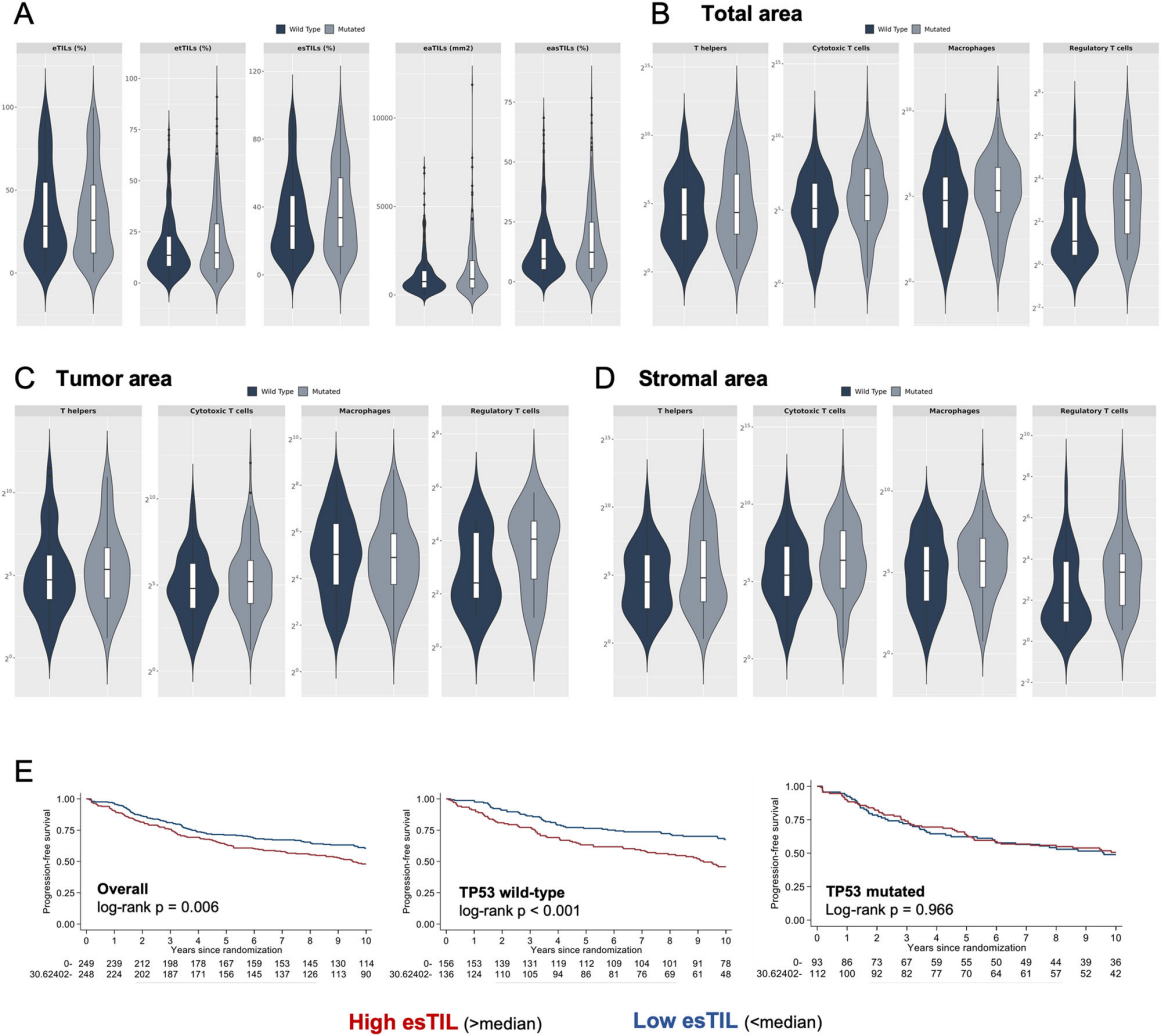
Composition of immune landscape according to *TP53* mutational status and prognostic correlates. **A** Distribution of digital TILs variables in patients with *TP53*-mutated versus *TP53* wild-type tumors; Expression of immune cell subpopulations according to *TP53* mutational status in the total (**B**), tumor (**C**) and stromal (**D**) areas, cell densities values are log2-transformed; **E** Kaplan-Meier curves on the prognostic effect of the interaction between esTILs and *TP53* mutational status (*p* = 0.028). Higher TILs were associated with worse PFS in patients with *TP53* wild-type but not in the *TP53*-mutated tumors.

Given that *TP53* mutational status was available for most patients included in EORTC10994/BIG1-00, we aimed to investigate its association with the composition of the immune infiltrate. To this end, we performed correlative analyses in the patient subgroup with known *TP53* status and available dTILs (*n* = 497) or multiplex immunofluorescence (*n* = 415) data. Stroma dTILs metrics were higher in the *TP53*-mutated patient subgroup versus the *TP53* wild-type in the whole population, but not within IHC-subtypes—although numerically higher in the *TP53*-mutated triple-negative group (Fig. 5A, Supplementary Table 6). When evaluating the distribution of immune cell subpopulations per *TP53* mutational status, we observed that cell densities of cytotoxic T-cells (at total, tumor and stroma areas), intra-tumoral T-regulatory cells, and stromal macrophages were significantly higher in the *TP53*-mutated patient subgroup (Fig. 5B–D, Supplementary Table 7). Similarly, PD-L1 expression on T-cells and macrophages was significantly enriched in the *TP53*-mutated patients, regardless of their localization. PD-1 expressing cells were also enriched in the *TP53*-mutated patients, mainly the intra-tumoral T-helpers and stromal cytotoxic T-cells and macrophages (Supplementary Table 7). Nevertheless, no difference was observed in NMS or entropy gradient score for interactions among tumor and immune cells according to *TP53* mutational status (Supplementary Figs. 5-6, Supplementary Table 8). Lastly, in an exploratory analysis, a significant interaction between esTIL and *TP53* mutational status was noted for PFS (*p* = 0.028), where higher esTIL abundance was prognostic for worse PFS only in patients with *TP53* wild-type tumors but not in mutant ones (Fig. 5E).

## Discussion

Breast cancer is a multifaceted ecosystem comprising various cell phenotypes and clones that may provide biological insights and hints towards biomarker development. The best studied immune markers are the assessment of TILs and PD-L1, which although prognostic in the early BC setting, they lack at this time clinical utility for routine use^1–5,11^. In addition, their static and visual evaluation cannot adequately grasp the complexity of immune infiltrate and the spatial distribution of tumor-host interactions. Toward this end, emerging powerful AI- and ML-based tools and spatial phenotypic quantitative immunohistochemistry/fluorescence technologies could facilitate comprehensive TME profiling^7,9,12^.

In this study, we investigated the immune cell composition and spatial interactions within the BC TME using ML-based and digital pathology algorithms and demonstrated their feasibility in early BC using small tissue input. By using a digital-assisted classifier on H&E-stained TMA, we showed that easTILs were associated with increased pCR rates, yet we could not demonstrate any significant correlation with long-term survival outcomes. Although this algorithm has been previously tested and validated in adjuvant setting for TNBC patients and demonstrated the prognostic correlation of high dTILs with improved survival^13^, inconsistent results have been reported in the neoadjuvant setting. In previous studies, both in HER2-negative^14^ and HER2+ disease^15^, easTILs correlated with pCR but not with long-term outcomes. On the other hand, a recent report using another AI-based dTILs algorithm on luminal tumors demonstrated a correlation with worse outcome -however no patient received neoadjuvant treatment^16^. Of note, a previous study using gene expression data from the EORTC 10994/BIG 1-00 trial reported that high expression of an 8-gene TILs-related signature correlated with increased pCR rates in ER-negative patients^17^. The results of the present study indicate that dTILs were associated with increased pCR in the ER+/HER2- subtype, however low patient numbers of patients in the other subtypes preclude any informative conclusions. Thus, it is unclear if these results could be attributed to biological or technical aspects. Regarding the latter, digital TILs evaluation could be prone to various analytical setbacks including interobserver variability, artifacts, algorithm training and tissue recognition, thus leading to discrepancies with the visual assessment and further impeding clinical utility^8,18^. The International Immuno-Oncology Biomarker Working Group has previously launched a set of recommendations on the computational TILs assessment, for overcoming the inherent limitations of visual TILs enumeration^19^. Furthermore, several versatile interpretable deep-learning algorithms for TILs evaluation have been recently reported i.e., MuTILs^20^ or the Histomic Prognostic Signature (HiPS)^21^, focusing jointly on tissue region and individual cell nuclei segmentation (compared to our cell nuclei-based classification approach), and outperforming the pathologist-based visual assessment in predicting survival outcomes. Given the increasing number of AI-based models generated for TILs assessment, a recent study compared the validity of ten AI models on TILs scoring, confirming discrepancies and variability in terms of mostly analytical rather than prognostic performance in patients with TNBC^22^. Therefore, future perspectives towards potential clinical implementation include the refinement of existing or design of next-generation AI models (i.e., foundation models)^23^, validation in prospective clinical studies and integration of computational image-based tools to other multi-omic data types for generation of comprehensive prognostic and predictive models of treatment response^24^.

In order to further investigate the immune cell composition at a spatial resolution, we performed multiplex immunofluorescence for T-cell and macrophage markers and for the expression of PD-1/PD-L1 checkpoints. Higher immune infiltration was seen in the TNBC subtype and similarly to dTILs analysis, CD8+ and CD4 + T-cells were correlated with increased pCR rates but not with long-term outcomes. Conflicting data have been reported regarding the association of single immune markers with pCR in early BC, highlighting thus the challenges of multiplex assays^9,25^. However, these multidimensional methods could provide further directions beyond the immune cell abundance, hence a growing body of literature is focusing on the spatial interactions among the different cells within the TME^6^. In the ARTEMIS trial on neoadjuvant chemotherapy for TNBC, a closer spatial proximity of T cells to cancer cells was associated with increased pCR rates^25^. Similar results on spatial immune infiltrate patterns have been recently reported in association with benefit to immunotherapy in TNBC^26,27^ and other tumor types^28,29^, further indicating that close interaction of tumor with activated immune cells is a major predictor of treatment response. In line with the aforementioned reports, we demonstrated here a higher and closer proximity of immune cells to cancer cells in TNBC patients that achieved pCR compared to those that did not respond to neoadjuvant chemotherapy treatment. Therefore, coupling deep-learning features with the spatial TME distribution in common predictive models could lead to improved patient stratification and treatment outcomes^30^.

Considering the previous reports on the link of the genetic determinants with antitumor immunity and that investigating the predictive role of *TP53* mutational status was a primary objective of the trial, we explored the potential effect of *TP53* mutation on TME. *TP53* has been shown to regulate the PD-1/PD-L1 axis in lung cancer studies, with IHC p53 positivity being associated with higher PD-L1 tumor cell expression^31,32^—a finding which was not confirmed in the case of breast cancer and *TP53* mutations^33^. On the other hand, it was recently shown that *TP53*-mutated BC exhibits significantly higher expression of chronic inflammation markers (i.e interferon signaling, CD8 + T-cell infiltration) and immune checkpoints, indicating that *TP53*mut-targeting compounds could restore effective immune surveillance^34^. In accordance with previous reports, we demonstrated an increased immune cell abundance (and respective checkpoint expression) in the *TP53*-mutated group of patients and especially in the TNBC subtype, possibly indicating that the presence of the mutation contributes to a more inflammatory phenotype. Moreover, in an exploratory analysis, we demonstrated a significant interaction between *TP53* mutational status and esTILs, with higher esTIL abundance being prognostic for worse PFS only in patients with *TP53* wild-type tumors. It is unclear if this prognostic effect could solely rely on the *TP53* biological effect or it reflects the association of increased TILs with more aggressive luminal B tumors^4,35^.

Although the presented results stem from a randomized phase III trial with long-term follow-up and highlight the advantages of automated and ML-based approaches, our study has several limitations needed to be addressed. A major limitation is the use of TMA, with cores of very small diameter (0.6 mm) which could have affected cellularity, immune cell abundance, tumor heterogeneity aspects and spatial tissue morphology analyses compared to whole tissue section^36^. The selection of representative tumor-rich areas for TMA construction and the inherent limitations of the used classifier could not distinguish between stromal and intratumoral TILs, thus affecting variable definitions and outcome correlations. The multicentric nature of the study could have also contributed to the tissue quality and TMA handling heterogeneity. Moreover, tumor phenotyping was performed on TMA and by using ≥1% cut-off for ER/PR positivity^37,38^; however, ER/PR cut-off ≥10% is used for positivity in some centers, based on previous studies demonstrating that patients with tumors with ER 1–9% and HER-2 negativity have mostly similar outcomes to those with ER ≤ 1%^39–41^. Although we showed that immune infiltrate correlated with chemosensitivity in the whole cohort, the relatively low number of patients within subgroups precluded definite conclusions in the exploratory analyses. Moreover, due to the exploratory nature of this study, no formal adjustment for multiplicity was performed. Lastly, at the time the trial was conducted, neoadjuvant chemotherapy alone was standard treatment. Therefore, any prognostic or predictive value of the immune infiltrate for the effect of newer neoadjuvant therapies (i.e., immunotherapy, anti-HER2) could not be evaluated.

In conclusion, our data indicate that ML-based algorithms could be used for characterizing the immune infiltrate in situ in early BC, which in turn contains prognostic information. Further studies are warranted in order to provide insights into TME cell phenotype features, interactions and underlying molecular determinants with the overall aim to improve prognostication and select candidates for future therapies.

## Methods

### Study design and patient cohort description

The present analysis is a translational substudy of the multicenter, international, randomized phase III EORTC 10994/BIG 1-00 clinical trial (Clinicaltrials.gov identifier NCT00017095). The study enrolled women (<71 years) with histologically-proven locally advanced, inflammatory, or large operable primary invasive BC who were candidates for neoadjuvant chemotherapy. Patients were randomly assigned in a 1:1 ratio to receive six cycles of taxane-based (docetaxel for three cycles followed by three cycles of epirubicin + docetaxel, T-ET; 928 patients) or non-taxane-based (5- fluorouracil, epirubicin, cyclophosphamide, FEC100 or tailored FEC; 928 patients) chemotherapy, prior to surgery. Post-surgery therapy included hormonal treatment and radiotherapy, according to the protocol guidelines. None of the patients received neoadjuvant trastuzumab or radiotherapy before surgery^10^. Patients who had signed informed consent, with available tissue microarray (TMA) and with IHC-based subtype defined per TMA were included in this translational study. The analyses are reported according to the Reporting Recommendations for Tumor Marker Prognostic Studies (REMARK) guidelines^42^ (Supplementary Table 9). The EORTC 10994/BIG 1-00 randomized phase III trial was registered at ClinicalTrials.gov (NCT00017095) and approved by the Ethics Committees in all participating centers (Supplementary Table 10). The proposed translational study has been reviewed and approved by the EORTC Translational Research Advisory Committee and Headquarters and the tissue material has been handled according to the signed material transfer agreement by the two organizations (Karolinska Institutet and EORTC). Only the tissue from the patients who have previously provided/signed informed consent for additional biologic research on their samples was included and analyzed. All patients have signed informed consent -prior registration- for inclusion in the trial and the assessment of p53 mutational status. The present study has been performed according to the Declaration of Helsinki, principles of Good Clinical Practice and was also approved by the Swedish Medical Product Agency and the Regional Ethical Committee in Stockholm (Dnr 01-387, Dnr 2005/472-32, Dnr 2005/738-32, Dnr 2008/897-32, Dnr 2013/954-32, Dnr 2018/540-32, Dnr 2021-05174, Dnr 2022-00657-02).

### Tissue sample preparation and histopathology assessment

Formalin-fixed paraffin-embedded (FFPE) tumor tissue blocks from the initial diagnostic biopsies (before initiation of neoadjuvant chemotherapy) have been collected from all patients. Hematoxylin & eosin (H&E)-stained slides were reviewed by a certified pathologist (G.M.) and representative tumor-rich areas were selected for TMA construction, performed at Institut Bergonié, Bordeaux, France. For each patient, up to three cores (0.6 mm in diameter each) were used for the TMA, as previously described^43^. TP53 mutational status analysis (assessed via a yeast functional assay and further validated through Sanger and next-generation sequencing) has been previously performed on baseline frozen tumor biopsies from all patients included in the study, with most (80%) having evaluable TP53 mutational status^10,43,44^. Tumor IHC-based phenotyping and biomarker interpretation were performed on TMA as follows: Estrogen receptor (ER) and progesterone receptor (PR) were defined as positive in case of ≥1% tumor cell expression; high Ki67 expression was defined as ≥14% of positive cells; human Epidermal Growth Factor Receptor 2 (HER2) was evaluated according to the ASCO/CAP 2013 recommendations and was considered positive if immunohistochemistry (IHC) 3+ or if IHC 2+ and ≥6 HER2 gene copies using in situ hybridization^43^. Tumors were classified into three subtypes as such: hormone receptor-positive/HER2-negative (HR+/HER2-negative), HER2-positive and triple negative (TN). Histological grade, type as well as pathological response (after neoadjuvant chemotherapy) were locally evaluated at each participating center.

### Digital evaluation of tumor-infiltrating lymphocytes

One FFPE section (4 μm thickness) was obtained from each TMA tissue block and stained with hematoxylin and eosin (H&E). H&E slides were digitized using the Nanozoomer 2.0-HT (Hamamatsu Photonics K.K.) platform at 20× magnification. TILs were subsequently enumerated using the digitally-assisted, image-based automated scoring CNN11 algorithm^13,45^. This previously trained and optimized classifier -which can be used with the open-source QuPath software- was used for detecting lymphocytes, tumor cells, stromal cells (e.g., fibroblasts) and “other” cells and for defining annotation and accumulative areas of each cell type (measured in mm^2^). Quality control of the tissue morphology (artifacts, necrosis) and cell segmentation was performed and subsequently five different TILs variables were derived from the different annotations as follows: i) eTILs (%) = 100 * (TILs/sum of tumor cells and TILs); ii) etTILs (%) = 100 * (TILs/All detected cells (i.e., tumor cells + fibroblasts + other cells)); iii) esTILs (%) = 100 * (TILs/Stromal cells (i.e TILs + fibroblasts + other cells)); iv) eaTILs (mm^2^) = TILs/tumor region areas analyzed; v) easTILs (%) = 100 * (sum of TILs area (mm^2^)/stroma area (mm^2^)), mirroring the definition provided by the International TILs Working Group guidelines)^1^. Intratumoral TILs were included in the respective calculations of all the aforementioned metrics.

### Multiplex immunofluorescence staining, image processing, and analysis

#### Staining

We performed multiplex immunofluorescence staining on FFPE TMA tumor tissue sections (thickness: 4 μm), using the Opal^TM^ 7-color Solid Tumor Immunology Kit (Akoya Biosciences, Marlborough, MA, USA), according to the manufacturer’s instructions. The automated Leica Bond RX^m^ system (Leica Biosystems, Buffalo Grove, IL, USA) was used for sequential staining with a panel of lymphocytic and macrophage markers using antibodies against CD4, CD8, PD-L1, PD-1, FoxP3, CD68. For the detection of epithelial tissue, a combination of antibodies against cytokeratin and E-cadherin was used, as previously described^36,46,47^. The list of antibodies, reagents, and experimental conditions used for the multiplex immunofluorescence staining are listed in Supplementary Table 11.

#### Imaging, image analysis, and quality control

The stained TMA were imaged using the Vectra® PolarisTM Automated Quantitative Pathology Imaging System (Akoya Biosciences, Marlborough, MA, USA) in multispectral mode at a resolution of 0,496 μm/pixel, which resulted in a total of 2350 multispectral images of size 0.93 mm × 0.7 mm. Each of the images was manually reviewed by three investigators (I.Z., A.M., C.B.) and curated to exclude artifacts, staining defects, and accumulation of immune cells in necrotic areas and intraglandular structures. Any discrepancies were resolved by a pathologist (A.Mez.). The number of images used for further analysis was 1414. The vendor-provided machine learning algorithm, implemented in the inForm^®^ image analysis software, was trained to split tissue into three categories: tumor compartment, stromal compartment, or blank areas. The training was performed on a selection of representative cores by providing a set of samples that was manually annotated. Cell segmentation was performed using DAPI nuclear staining. The perinuclear region at 7 pixels from the nuclear border was considered as the cytoplasm area.

#### Cell phenotyping

The cell phenotyping function of the inForm® image analysis software was used to manually define a representative subset of cells positive to expression of each of the markers and a subset of cells negative to all markers (also considering the rare immune phenotypes). The marker expression was evaluated in the cytoplasm of the segmented cells as the number of photons, normalized to exposure time. Exceptions were made for i) FoxP3 (expressed in nuclei and thus its signal was analyzed in the nuclear region of segmented cells) and ii) CD4 marker, which demonstrated more diffuse staining (probably due to epitope instability) often covering also nuclei regions, and thus, the average marker expression level in total cell region (nucleus + cytoplasm) was used^48^. Intensity cutoffs for the markers were determined in the R programming environment (version 3.6.0). The marker-specific cutoffs were defined by analyzing the distributions of the positive intensities for the marker in the manually-annotated cells and by controlling for the background levels visually and in the negatively annotated cells. The empirical cumulative distribution function was used for visual control. The minimal cutoff was selected as minimal recorded signal level (among the manually annotated cells) if the empirical cumulative distribution demonstrated steep monotonous tendency. In case of individual low-level outliers, they were considered as negative. In case of prominent “kneepoint” in the graph the expression of the marker was back-controlled by reviewing the annotated images and controlling for the marker expression. Additionally, for each of the markers the images were screened for the areas of non-specific staining which were considered as background. Due to the high fraction of CK-positive cells in the entire cohort, a different approach was used to define its cutoff. The complete dataset, containing 1,790,165 cells, was analyzed to evaluate the distribution of CK expression, measured as marker mean expression in cell cytoplasm. The distribution had two peaks, reflecting non-CK cells (low expression) and CK-positive cells (high expression) and Otsu algorithm^49^ was used to find minima between two groups.

#### Cell classification

Using the established cutoff levels, every cell was characterized as positive or negative for each marker in the panel. These data were used to classify the cell and define its immune subtype as illustrated in Supplementary Table 12. Multiple TMA cores from the same tumor/patient were merged and cell abundance was normalized to the total tissue area. Cell subclasses were quantified and normalized to tissue area thus resulting in final metric called “cell density” (units per mm^2^), which was calculated in total tissue area, stroma, and tumor regions for each cell subclass. For illustration purposes, the cell density values were log2- transformed. The multispectral imaging/analysis workflow is depicted in Supplementary Fig. 7.

### Spatial image analysis and topographical interactions

The previously described Spatial Image Analysis of Tissues (SPIAT) toolkit^50^ was utilized to facilitate the detection of cellular interactions among immune cells (including regulatory T-cells, cytotoxic T-cells, macrophages, and T-helpers and the respective expression of PD-L1/PD-1 checkpoints as reference cells) with tumor cells (target cells) and spatial cell localization. The applied metrics included the normalized mixing score and the entropy gradient which both evaluated the co-localization of immune and tumor cells. The analysis was performed on single reconstructed images from multiple patient-specific TMA stitched with a proper distance between them to avoid potential overlaps. Normalized mixing score (NMS) and the “aggregated entropy” were calculated as a function of different radii around the reference cells (immune cells here), varying from 50 up (close-distance interactions) to 600 μm to cover the whole TMA area with a step of 50μm. The NMS uses a normalization scale factor -accounting for cell number- and receives a numerical value according to the following formula, described in SPIAT: NMS = number of interactions between reference and target cells × (total number of reference cells – 1)/2 × number of interactions between reference cell types x total number of target cells. The higher the score, the higher the degree of co-localization between immune and tumor cells. Given that cell number and types in TMAs are fixed, the definition of entropy entails mainly the variability in cell number rather than degree of randomness. Immune cells were selected as reference, target cells were identified for each selected radius around the reference cells and entropy scores were subsequently calculated for each radius (“aggregated entropy” as described in the SPIAT toolkit)^50^. Higher entropy scores correspond to high cell colocalization between reference and target cells and vice versa for low entropy scores (unbalanced cell types – low colocalization). Next, patient-specific gradient entropy curves were generated from the “aggregated entropy” values along the different radii (for illustration purposes all curves were graphically displayed following a 10μm radius step) and classified either as “Attraction-like” or “Repulsion-like” patterns according to the slope of the curve. If the slope was negative close to the lowest radius, the pattern of interaction was defined as ”Attraction”, indicating higher aggregated entropy values close to reference cells, thus high colocalization. If the slope was positive close to the lowest radius, the pattern of interaction is defined as ”Repulsion”, indicating higher aggregated entropy values far from reference cells, thus low colocalization. In case of no difference on entropy values across the radii, there was a zero slope^50^. The aforementioned analyses were performed in the R programming environment.

### Outcomes definition and statistical analysis

Pathologic complete response (pCR) was defined as in the original publication of the primary efficacy analysis of the trial as the absence of residual invasive cancer (or very few scattered cancer cells left), with or without residual DCIS and with negative axillary lymph nodes (ypT0/is ypN0). Non-pCR patients included those who presented with tumor progression on neoadjuvant chemotherapy or who did not undergo surgery^43^. Patients with missing information were excluded from the analysis. Progression-free survival (PFS), which was the primary endpoint of the trial^10^, was defined as the time from randomization to progression on neoadjuvant therapy, locoregional relapse (invasive cancer), first distant metastasis, invasive contralateral breast cancer or death from any cause, whichever occurred first. Second primary invasive non-breast cancers, DCIS or LCIS (ipsilateral or contralateral) were not considered as events.

Continuous outcomes were tested using the Wilcoxon rank-sum or Kruskal–Wallis test while binary outcomes with Pearson’s chi-squared or Fisher’s exact test. The Spearman’s rank correlation coefficient was used for correlations between continuous variables. The different immune cell subpopulations and digital metrics derived by dTILs and multiplex immunofluorescence were correlated to pCR using univariate and multivariable logistic regression models. Median follow-up was calculated using the reverse Kaplan-Meier method. Survival (PFS) was calculated using the Kaplan-Meier method and differences in PFS were calculated with the log-rank test. Time to failure was modeled using proportional hazard regression. Logistic regression and Cox regression models are presented as odds ratios and hazard ratios, respectively, with the respective 95% confidence intervals and Wald p-values. Multivariable models were adjusted for tumor size, nodal status and treatment, and stratified for subtype since subtype violated proportionality of hazards. All p-values were two-sided with 5% as the level of significance. Adjustment for multiplicity was not performed. All analyses were performed the Stata software (v. 17, StataCorp, College Station, TX, USA).

## Supplementary information

Supplementary Material

## Data Availability

Data shall be shared according to the EORTC data release policy (http://www.eortc.org/datasharing/).

## Code availability

The code for the multiplex immunofluorescence-based data analysis could be found in the following link: https://github.com/gmanikis/EORTC_NPJ_BC/tree/main.
