# Supplementary material for: Machine learning-based spatial characterization of tumor-immune microenvironment in the EORTC 10994/BIG 1-00 early breast cancer trial
Source: NPJ Breast Cancer. 2025 Mar 7;11:23. doi: 10.1038/s41523-025-00730-1 (PMC11889191; doi:10.1038/s41523-025-00730-1)
Supplement: Supplementary file 1 — Supplementary Material [file 41523_2025_730_MOESM1_ESM.pdf]

Supplementary Material

Machine learning-based spatial characterization of tumor-immune microenvironment in the EORTC 10994/BIG 1-00 early breast cancer trial

Ioannis Zerdes*[1,2], Alexios Matikas*[1,2], Artur Mezheyeuski[3,4#], Georgios Manikis[1,5#], Balazs Acs[1,6], Hemming Johansson[1], Ceren Boyaci[1,6], Caroline Boman[1,2], Coralie Poncet[7], Michail Ignatiadis[8], Yalai Bai[9,10], David L. Rimm[9,10], David Cameron[11], Hervé Bonnefoi[12], Jonas Bergh[1,2], Gaetan Mac Grogan[13], Theodoros Foukakis[1,2]

Authors' affiliations:

1. Department of Oncology-Pathology, Karolinska Institutet, Stockholm, Sweden
2. Breast Center, Theme Cancer, Karolinska Comprehensive Cancer Center and University Hospital, Stockholm, Sweden
3. Department of Immunology, Genetics, and Pathology, Uppsala University, Uppsala, Sweden
4. Molecular Oncology Group, Vall d'Hebron Institute of Oncology, Barcelona, Spain
5. Computational BioMedicine Laboratory (CBML), Foundation for Research and Technology-Hellas (FORTH), Heraklion, Greece
6. Department of Clinical Pathology and Cancer Diagnostics, Karolinska University Hospital, Stockholm, Sweden
7. European Organisation for Research and Treatment of Cancer Headquarters, Brussels, Belgium

8. Department of Medical Oncology, Institut Jules Bordet and L'Université Libre de Bruxelles (U.L.B), Brussels; Academic Trials Promoting Team (ATPT), Institut Jules Bordet, Brussels, Belgium

9. Department of Pathology, Yale School of Medicine, New Haven, CT, USA

10. Yale Cancer Center, Yale School of Medicine, New Haven, CT, USA

11. Edinburgh University Cancer Centre, Institute of Genetics and Cancer, University of Edinburgh, Edinburgh, UK

12. Department of Medical Oncology, Institut Bergonié Unicancer, INSERM U1312, Université de Bordeaux, Bordeaux, France

13. Department of Biopathology, Institut Bergonié Unicancer, INSERM U1312, Bordeaux, France

* Denotes co-first authorship

# Denotes co-second authorship

Total number of supplementary-only material: Supplementary Tables: 12, Supplementary Figures: 7

Supplementary Tables & Figures Legends

Supplementary Table 1. Baseline demographic characteristics for patients included in this study, eligible for digital TILs and multiplex immunofluorescence analysis in EORTC 10994/BIG 1-00 trial

Supplementary Table 2. Distribution of digital TILs in the whole population (n=587) and within IHC-based subtypes

Supplementary Table 3. Distribution (cell densities=number of positive cells/mm^2) of multiplex immune cell subpopulations (single and double markers) in the whole population at the total (n=478), intra-tumoral (n=468) and stromal (n=478) areas

Supplementary Table 4. Distribution of cell densities (i.e. number of positive cells/mm^2) of multiplex fluorescence immune cell subpopulations (single and double expression markers) within IHC-based subtypes in the total area (n=478) and in the intra-tumoral (n=468) and stromal (n=478) areas

Supplementary Table 5. Distribution of the normalized mixing score (median value) spatial metric of cancer-immune cells interaction (derived from the multiplex fluorescence analysis) according to the pCR status in the triple-negative subtype

Supplementary Table 6. Distribution of digital TILs (n=497, mean) according to the *TP53* mutational status in the whole population

Supplementary Table 7. Distribution (cell densities=number of positive cells/mm^2) of immune cell subpopulations (single and double markers, n=415 for total and for stromal area, n=407 for intra-tumoral area) according to the *TP53* mutational status

Supplementary Table 8. Distribution of the normalized mixing score (median value) spatial metric of cancer-immune cells interaction (derived from the multiplex fluorescence analysis) according to the *TP53* mutational status in the whole population

Supplementary Table 9. REMARK checklist

Supplementary Table 10. List of all participating centers in the clinical study

Supplementary Table 11. List of antibodies and experimental conditions used for the multiplex immunofluorescence method

Supplementary Table 12. Cell classification based on the markers derived from the multiplex immunofluorescence method

Supplementary Figure 1. Forest plots on prognostic effect of digital TILs on progression-free survival (PFS); A. Univariate model; B. Multivariable model, adjusted for tumor size, nodal status, treatment and stratified by subtype

Supplementary Figure 2. Correlation matrix for digital TILs and multiplex immunofluorescence immune subpopulations (n = 387) for (A) single marker and (B) double marker expression in total area;

Supplementary Figure 3. Forest plots on prognostic effect of single and double multiplex markers on progression-free survival (PFS); A. Univariate model; B. Multivariable model, adjusted for tumor size, nodal status, treatment and stratified by subtype

Supplementary Figure 4. Entropy gradient slopes according to pCR status in triple-negative subtype for interactions of tumor cells with all immune cells (A), cytotoxic T-cells (B), T-helpers (C), T-regulatory cells (D), macrophages (E); each curve represent the median of all radii for the specific cell interactions in an attraction- or repulsion-like pattern

Supplementary Figure 5. Normalized mixing score according to *TP53* mutational status (representative images of the spatial distributions, A) in the overall population patients for interactions of tumor cells with all immune cells (B), cytotoxic T-cells (C), T-regulatory cells (D), macrophages (E) and T-helpers (F)

Supplementary Figure 6. Entropy gradient slopes slopes according to *TP53* mutational status in the overall population patients for interactions of tumor cells with all immune cells (A), cytotoxic T-cells (B), T-helpers (C), T-regulatory cells (D), macrophages (E); each curve represent the median of all radii for the specific cell interactions in an attraction- or repulsion-like pattern

Supplementary Figure 7. Workflow for the multiplex immunofluorescence multispectral image analysis

Supplementary Table 1. Baseline demographic characteristics for patients included in this study, eligible for digital TILs and multiplex immunofluorescence analysis in EORTC 10994/BIG 1-00 trial

	**Digital TILs population (N=587)**	**mIHC population (N=478)**
**Age (years)**		
<50	348 (59.3%)	281 (58.8%)
≥ 50	239 (40.7%)	197 (41.2%)
**Stage**		
Locally advanced/Inflam	118 (20.1%)	78 (16.3%)
Large operable	469 (79.9%)	400 (83.7%)
**p53 status**		
Wild type	292 (49.7%)	242 (50.6%)
Mutated	205 (34.9%)	164 (34.3%)
Unknown	90 (15.3%)	72 (15.1%)
**Menopausal status**		
Pre-menopausal	347 (59.1%)	283 (59.2%)
Post-menopausal	239 (40.7%)	192 (40.2%)
Unknown	1 (0.2%)	3 (0.6%)
**Performance status**		
0	549 (93.5%)	447 (93.5%)
1-2	38 (6.5%)	31 (6.5%)
**Clinical tumor status**		
T1-T2	329 (56.0%)	268 (56.1%)
T3- T4	258 (44.0%)	210 (43.9%)
**Clinical nodal status**		
N0	261 (44.5%)	230 (48.1%)
N1	294 (50.1%)	229 (47.9%)
N2	28 (4.8%)	17 (3.6%)
N3	4 (0.7%)	2 (0.4%)
**Histological grade**		
G1	41 (7.0%)	34 (7.1%)
G2	288 (49.1%)	239 (50.0%)
G3	206 (35.1%)	156 (32.6%)
NA/Unknown	50 (8.5%) / 2 (0.3%)	47 (9.8%) / 2 (0.4%)
**Endocrine treatment**		
None	153 (26.1%)	100 (20.9%)
Tam/AI	434 (73.9%)	378 (79.1%)
**Simplified subtype (based on TMA)**		
HR+/HER2-	371 (63.2%)	323 (67.6%)
HER2+	129 (22.0%)	94 (19.7%)
Triple-negative	87 (14.8%)	61 (12.8%)
**Treatment**		
FEC	297 (50.6%)	230 (48.1%)
T-ET	290 (49.4%)	248 (51.9%)

Supplementary Table 2. Distribution of digital TILs in the whole population (n=587) and within IHC-based subtypes

	**Overall (N=587)**	**HER2+ (N=129)**	**HR+HER2- (N=371)**	**TN (N=87)**	**P-value***
**eTILs (%)**					
Mean (SD)	36.0 (26.3)	37.0 (26.1)	36.2 (26.7)	33.9 (25.1)	0.714
Median [Min, Max]	30.0 [0.0883, 100]	34.7 [0.466, 93.4]	28.2 [0.0883, 100]	30.8 [0.674, 100]	
**etTILs (%)**					
Mean (SD)	19.1 (16.0)	19.8 (16.8)	17.8 (14.8)	23.1 (19.0)	0.096
Median [Min, Max]	14.3 [0.0881, 91.0]	13.7 [0.217, 75.0]	13.7 [0.0881, 91.0]	19.1 [0.639, 80.3]	
**esTILs (%)**					
Mean (SD)	35.7 (24.1)	34.1 (22.9)	33.6 (23.0)	47.0 (27.2)	**<0.001**
Median [Min, Max]	30.6 [0.124, 99.9]	31.2 [0.389, 97.7]	28.1 [0.124, 99.9]	43.0 [3.33, 97.7]	
**eaTILs (mm2)**					
Mean (SD)	1250 (1360)	1260 (1330)	1120 (1230)	1800 (1760)	**0.004**
Median [Min, Max]	802 [3.08, 11900]	819 [7.26, 6880]	750 [3.08, 11900]	1270 [46.5, 7750]	
**easTILs (%)**					
Mean (SD)	15.3 (13.8)	15.1 (14.0)	13.7 (12.2)	22.3 (17.2)	**<0.001**
Median [Min, Max]	11.1 [0.0177, 76.7]	11.6 [0.0965, 66.3]	9.85 [0.0177, 76.7]	18.0 [1.31, 69.6]	

*p-value corresponds to the comparison among the three different subtypes (Kruskal-Wallis test)

Supplementary Table 3. Distribution (cell densities=number of positive cells/mm^2) of multiplex immune cell subpopulations (single and double markers) in the whole population at the total (n=478), intra-tumoral (n=468) and stromal (n=478) areas

	Mean (SD)	Median [min, max]
**T helpers (CD4+)**		
Total area	78.7 (324)	0 [0, 3580]
Intra-tumoral	54.7 (286)	0 [0, 3760]
Stromal	88.6 (387)	0 [0, 5460]
**Cytotoxic T-cells (CD8+)**		
Total area	139 (384)	32.6 [0, 5520]
Intra-tumoral	69.5 (264)	11.6 [0, 4330]
Stromal	168 (428)	38.2 [0, 5830]
**Macrophages (CD68+)**		
Total area	59.3 (118)	20.2 [0, 1610]
Intra-tumoral	34.3 (62.9)	7.29 [0, 439]
Stromal	84.3 (203)	19.4 [0, 3210]
**Regulatory T-cells (FOXP3+)**		
Total area	2.34 (10.7)	0 [0, 108]
Intra-tumoral	1.04 (6.87)	0 [0, 113]
Stromal	3.29 (17.1)	0 [0, 231]
**PD-L1+ CD4+ T-cells**		
Total area	42.1 (253)	0 [0, 3220]
Intra-tumoral	27.9 (210)	0 [0, 3650]
Stromal	49.3 (309)	0 [0, 4810]
**PD-L1+ CD8+ T-cells**		
Total area	29.4 (180)	0 [0, 2950]
Intra-tumoral	19.4 (160)	0 [0, 2720]
Stromal	34.8 (191)	0 [0, 3150]
**PD-L1+ CD68+ cells**		
Total area	12.1 (71.3)	0 [0, 1320]
Intra-tumoral	5.14 (27.0)	0 [0, 428]
Stromal	18.8 (131)	0 [0, 2630]
**PD-L1+ Regulatory T-cells**		
Total area	1.29 (7.38)	0 [0, 90.4]
Intra-tumoral	0.619 (5.74)	0 [0, 110]
Stromal	1.91 (12.9)	0 [0, 193]
**PD-1+ CD4+ T-cells**		
Total area	18.9 (89.5)	0 [0, 883]
Intra-tumoral	10.1 (72.9)	0 [0, 1210]
Stromal	22.9 (116)	0 [0, 1740]
**PD-1+ CD8+ T-cells**		
Total area	16.9 (78.8)	16.9 (78.8)
Intra-tumoral	14.1 (136)	0 [0, 2770]
Stromal	18.8 (79.9)	0 [0, 945]
**PD-1+ CD68+ macrophages**		
Total area	3.23 (24.2)	0 [0, 468]
Intra-tumoral	1.18 (8.34)	0 [0, 117]
Stromal	5.44 (51.2)	0 [0, 950]
**PD-1+ Regulatory T-cells**		
Total area	0.630 (4.06)	0 [0, 62.8]
Intra-tumoral	0.268 (2.55)	0 [0, 47.8]
Stromal	0.908 (6.52)	0 [0, 92.4]

*p-value corresponds to the comparison among the three different subtypes (Kruskal-Wallis test)

Supplementary Table 4. Distribution of cell densities (i.e. number of positive cells/mm^2) of multiplex fluorescence immune cell subpopulations (single and double expression markers) within IHC-based subtypes in the total area (n=478) and in the intra-tumoral (n=468) and stromal (n=478) areas

	**HER2+ (n=94)**	**HR+/HER2- (n=323)**	**TN (n=61)**	**p-value**
**T helpers (CD4+)**				
**Total**				
Mean (SD)	102 (401)	52.5 (259)	182 (456)	<0.001
Median [Min, Max]	2.62 [0, 3580]	0 [0, 3530]	15.2 [0, 3030]	
**Intra-tumoral**				
Mean (SD)	43.2 (216)	42.4 (230)	139 (538)	<0.001
Median [Min, Max]	0 [0, 1970]	0 [0, 2860]	4.60 [0, 3760]	
**Stromal**				
Mean (SD)	127 (586)	51.0 (269)	228 (492)	<0.001
Median [Min, Max]	2.14 [0, 5460]	0 [0, 3800]	11.6 [0, 2680]	
**Cytotoxic T-cells (CD8+)**				
**Total**				
Mean (SD)	176 (334)	113 (375)	217 (483)	0.038
Median [Min, Max]	51.8 [0, 1690]	29.7 [0, 5520]	64.1 [0, 3090]	
**Intra-tumoral**				
Mean (SD)	54.5 (132)	54.6 (150)	173 (631)	0.114
Median [Min, Max]	11.1 [0, 720]	11.2 [0, 1300]	21.2 [0, 4330]	
**Stromal**				
Mean (SD)	221 (424)	133 (405)	268 (525)	0.009
Median [Min, Max]	66.2 [0, 2170]	32.1 [0, 5830]	77.6 [0, 3500]	
**Macrophages (CD68+)**				
**Total**				
Mean (SD)	71.8 (179)	48.9 (92.1)	94.7 (119)	0.001
Median [Min, Max]	22.0 [0, 1610]	15.9 [0, 794]	55.0 [0, 528]	
**Intra-tumoral**				
Mean (SD)	31.1 (64.1)	32.1 (57.1)	51.3 (85.7)	0.014
Median [Min, Max]	0 [0, 292]	8.24 [0, 376]	22.1 [0, 439]	
**Stromal**				
Mean (SD)	109 (344)	65.9 (135)	143 (200)	<0.001
Median [Min, Max]	19.9 [0, 3210]	16.4 [0, 1080]	89.5 [0, 1060]	
**Regulatory T-cells (FOXP3+)**				
**Total**				
Mean (SD)	2.58 (11.7)	0.75 (4.07)	10.4 (22.9)	<0.001
Median [Min, Max]	0 [0, 108]	0 [0, 65.0]	0 [0, 104]	
**Intra-tumoral**				
Mean (SD)	0.15 (0.93)	0.21 (1.88)	6.89 (17.9)	<0.001
Median [Min, Max]	0 [0, 7.82]	0 [0, 26.2]	0 [0, 113]	
**Stromal**				
Mean (SD)	4.29 (24.1)	0.98 (4.74)	14.0 (33.8)	<0.001
Median [Min, Max]	0 [0, 231]	0 [0, 69.8]	0 [0, 184]	
**PD-L1+ CD4+ T-cells**				

**Total**				
Mean (SD)	63.0 (342)	20.1 (175)	126 (389)	<0.001
Median [Min, Max]	0 [0, 3220]	0 [0, 3050]	4.12 [0, 2750]	
**Intra-tumoral**				
Mean (SD)	30.2 (196)	12.0 (83.9)	110 (498)	<0.001
Median [Min, Max]	0 [0, 1840]	0 [0, 1260]	0 [0, 3650]	
**Stromal**				
Mean (SD)	81.8 (504)	19.4 (187)	157 (401)	<0.001
Median [Min, Max]	0 [0, 4810]	0 [0, 3270]	0 [0, 2310]	
**PD-L1+ CD8+ T-cells**				
**Total**				
Mean (SD)	32.5 (127)	18.3 (168)	83.6 (280)	<0.001
Median [Min, Max]	1.37 [0, 901]	0 [0, 2950]	6.19 [0, 1850]	
**Intra-tumoral**				
Mean (SD)	12.5 (53.9)	7.29 (39.1)	95.6 (432)	<0.001
Median [Min, Max]	0 [0, 466]	0 [0, 419]	0 [0, 2720]	
**Stromal**				
Mean (SD)	38.5 (151)	21.0 (180)	102 (274)	<0.001
Median [Min, Max]	0.54 [0, 1030]	0 [0, 3150]	4.14 [0, 1800]	
**PD-L1+ CD68+ cells**				
**Total**				
Mean (SD)	21.5 (138)	5.33 (28.1)	33.8 (75.3)	<0.001
Median [Min, Max]	0 [0, 1320]	0 [0, 418]	2.00 [0, 356]	
**Intra-tumoral**				
Mean (SD)	4.60 (22.3)	2.53 (13.8)	20.0 (61.6)	<0.001
Median [Min, Max]	0 [0, 195]	0 [0, 185]	0 [0, 428]	
**Stromal**				
Mean (SD)	37.3 (272)	6.98 (34.1)	53.0 (116)	<0.001
Median [Min, Max]	0 [0, 2630]	0 [0, 449]	3.05 [0, 508]	
**PD-L1+ Regulatory T-cells**				
**Total**				
Mean (SD)	1.73 (9.59)	0.274 (2.81)	5.96 (14.8)	<0.001
Median [Min, Max]	0 [0, 90.4]	0 [0, 48.0]	0 [0, 76.9]	
**Intra-tumoral**				
Mean (SD)	0.065 (0.45)	0.082 (1.47)	4.37 (15.4)	<0.001
Median [Min, Max]	0 [0, 3.74]	0 [0, 26.2]	0 [0, 110]	
**Stromal**				
Mean (SD)	3.03 (20.0)	0.318 (3.06)	8.63 (24.2)	<0.001
Median [Min, Max]	0 [0, 193]	0 [0, 51.6]	0 [0, 159]	
**PD-1+ CD4+ T-cells**				
**Total**				
Mean (SD)	33.3 (130)	9.91 (59.1)	44.7 (131)	<0.001
Median [Min, Max]	0 [0, 883]	0 [0, 863]	1.17 [0, 670]	
**Intra-tumoral**				
Mean (SD)	6.92 (23.5)	5.21 (33.6)	41.5 (186)	<0.001
Median [Min, Max]	0 [0, 144]	0 [0, 404]	0 [0, 1210]	
**Stromal**				
Mean (SD)	44.4 (202)	10.9 (64.9)	53.2 (140)	<0.001

Median [Min, Max]	0 [0, 1740]	0 [0, 969]	0 [0, 664]	
**PD-1+ CD8+ T-cells**				
**Total**				
Mean (SD)	30.9 (91.7)	8.61 (58.4)	38.9 (130)	<0.001
Median [Min, Max]	0 [0, 593]	0 [0, 945]	2.32 [0, 867]	
**Intra-tumoral**				
Mean (SD)	8.76 (38.6)	6.18 (41.2)	64.8 (366)	<0.001
Median [Min, Max]	0 [0, 314]	0 [0, 475]	0 [0, 2770]	
**Stromal**				
Mean (SD)	38.7 (110)	9.02 (59.2)	40.2 (107)	<0.001
Median [Min, Max]	0 [0, 593]	0 [0, 945]	0 [0, 583]	
**PD-1+ CD68+ macrophages**				
**Total**				
Mean (SD)	6.87 (48.5)	1.23 (6.25)	8.20 (27.1)	<0.001
Median [Min, Max]	0 [0, 468]	0 [0, 83.7]	0 [0, 194]	
**Intra-tumoral**				
Mean (SD)	1.08 (6.03)	0.49 (3.13)	5.05 (20.8)	<0.001
Median [Min, Max]	0 [0, 53.1]	0 [0, 38.6]	0 [0, 117]	
**Stromal**				
Mean (SD)	12.5 (98.1)	1.37 (7.18)	16.1 (73.1)	<0.001
Median [Min, Max]	0 [0, 950]	0 [0, 96.3]	0 [0, 566]	
**PD-1+ Regulatory T-cells**				
**Total**				
Mean (SD)	1.09 (5.08)	0.125 (0.718)	2.60 (9.11)	<0.001
Median [Min, Max]	0 [0, 42.8]	0 [0, 9.30]	0 [0, 62.8]	
**Intra-tumoral**				
Mean (SD)	0.086 (0.82)	0.053 (0.60)	1.70 (6.85)	<0.001
Median [Min, Max]	0 [0, 7.82]	0 [0, 9.17]	0 [0, 47.8]	
**Stromal**				
Mean (SD)	1.70 (9.87)	0.158 (0.91)	3.65 (13.1)	<0.001
Median [Min, Max]	0 [0, 92.4]	0 [0, 9.32]	0 [0, 90.1]	

*p-value corresponds to the comparison among the three different subtypes (Kruskal-Wallis test)

Supplementary Table 5. Distribution of the normalized mixing score (median value) spatial metric of cancer-immune cells interaction (derived from the multiplex fluorescence analysis) according to the pCR status in the triple-negative subtype

**Radius**	**Cytotoxic T-cells**		**Immune cells***		**T-helpers**		**T-regulatory cells**		**Macrophages**	
	**pCR**	**non-pCR**	**pCR**	**non-pCR**	**pCR**	**non-pCR**	**pCR**	**non-pCR**	**pCR**	**non-pCR**
**50**	0.14	0.10	0.38	0.23	0.01	0.11	0.18	0.27	0.38	0.25
**100**	0.44	0.26	0.34	0.63	0.16	0.18	0.24	0.50	0.69	0.46
**150**	0.41	0.29	0.42	0.78	0.18	0.25	0.55	0.60	0.83	0.58
**200**	0.53	0.36	0.50	0.84	0.22	0.28	0.62	0.66	0.82	0.65
**250**	0.69	0.40	0.56	0.83	0.26	0.36	0.68	0.65	0.86	0.68
**300**	0.70	0.42	0.61	0.84	0.31	0.42	0.66	0.65	0.86	0.77
**350**	0.87	0.48	0.65	0.91	0.35	0.46	0.60	0.70	0.92	0.80
**400**	0.90	0.51	0.67	0.94	0.39	0.49	0.62	0.75	0.94	0.81
**450**	0.92	0.53	0.70	0.95	0.41	0.53	0.72	0.77	0.98	0.82
**500**	0.95	0.55	0.71	0.97	0.42	0.57	0.77	0.77	0.97	0.82
**550**	0.97	0.55	0.71	0.96	0.41	0.57	0.79	0.75	0.97	0.82
**600**	0.99	0.55	0.70	0.95	0.42	0.57	0.80	0.75	0.97	0.82

*Immune cells include cytotoxic T-cells, T-helpers, T-regulatory cells, macrophages and the respective co-expression of PD-L1/PD-1 checkpoints in each of these cell subsets

Supplementary Table 6. Distribution of digital TILs (n=497, mean) according to the *TP53* mutational status in the whole population

**Digital TILs** [mean (SD)]	**TP53 wild-type**	**TP53 mutated**	**p-value***
**Overall population**	n = 292	n = 205	
**eTILs (%)**			
Mean (SD)	36.2 (26.9)	36.2 (26.8)	0.99
Median [Min, Max]	28.3 [0.0883, 100]	31.8 [0.466, 100]	
**etTILs (%)**			
Mean (SD)	17.7 (14.7)	20.6 (17.5)	0.2
Median [Min, Max]	13.5 [0.0881, 75.0]	14.7 [0.217, 91.0]	
**esTILs (%)**			
Mean (SD)	33.8 (23.3)	38.4 (25.0)	**0.05**
Median [Min, Max]	28.7 [0.545, 97.7]	33.7 [0.389, 99.9]	
**eaTILs (mm^2)**			
Mean (SD)	1080 (1110)	1440 (1580)	**0.032**
Median [Min, Max]	749 [7.20, 7190]	899 [7.26, 11900]	
**easTILs (%)**			
Mean (SD)	13.6 (12.3)	17.4 (15.1)	**0.01**
Median [Min, Max]	9.58 [0.135, 68.4]	12.3 [0.096, 76.7]	
**HER2+ (IHC-based subtype)**	n = 48	n = 58	
**eTILs (%)**			
Mean (SD)	35.6 (26.4)	38.7 (27.7)	0.538
Median [Min, Max]	30.8 [0.716, 91.0]	37.7 [0.466, 93.4]	
**etTILs (%)**			
Mean (SD)	21.3 (18.9)	19.3 (16.1)	0.78
Median [Min, Max]	13.9 [0.538, 75.0]	12.8 [0.217, 63.2]	
**esTILs (%)**			
Mean (SD)	35.8 (24.4)	33.3 (22.2)	0.572
Median [Min, Max]	34.6 [2.12, 93.1]	29.5 [0.389, 95.5]	
**eaTILs (mm^2)**			
Mean (SD)	1370 (1490)	1250 (1300)	0.699
Median [Min, Max]	897 [29.4, 6880]	767 [7.26, 6160]	
**easTILs (%)**			
Mean (SD)	16.3 (15.2)	14.8 (13.8)	0.547
Median [Min, Max]	13.1 [0.633, 63.3]	11.0 [0.096, 66.3]	
**HR+/HER2- (IHC-based subtype)**	n = 224	n = 97	
**eTILs (%)**			
Mean (SD)	36.8 (26.9)	36.0 (27.9)	0.642
Median [Min, Max]	28.5 [0.0883, 100]	29.1 [0.792, 100]	
**etTILs (%)**			
Mean (SD)	16.8 (13.1)	19.8 (17.3)	0.509
Median [Min, Max]	13.5 [0.0881, 65.3]	15.2 [0.681, 91.0]	
**esTILs (%)**			
Mean (SD)	32.5 (22.6)	36.8 (24.0)	0.134
Median [Min, Max]	25.9 [0.545, 93.8]	33.0 [2.99, 99.9]	
**eaTILs (mm^2)**			

Mean (SD)	984 (915)	1340 (1570)	0.163
Median [Min, Max]	724 [7.20, 5090]	841 [40.7, 11900]	
**easTILs (%)**			
Mean (SD)	12.5 (10.7)	15.9 (13.8)	0.057
Median [Min, Max]	9.36 [0.135, 57.7]	11.1 [1.07, 76.7]	
**TN (IHC-based subtype)**	n = 20	n = 50	
**eTILs (%)**			
Mean (SD)	30.9 (28.5)	33.7 (23.4)	0.42
Median [Min, Max]	18.9 [2.26, 100]	32.2 [0.674, 87.1]	
**etTILs (%)**			
Mean (SD)	19.4 (18.2)	23.7 (19.3)	0.298
Median [Min, Max]	14.1 [2.21, 70.0]	19.8 [0.639, 80.3]	
**esTILs (%)**			
Mean (SD)	44.0 (27.2)	47.6 (27.8)	0.735
Median [Min, Max]	37.1 [6.68, 97.7]	43.8 [3.33, 93.3]	
**eaTILs (mm^2)**			
Mean (SD)	1450 (1700)	1880 (1830)	0.232
Median [Min, Max]	925 [78.9, 7190]	1320 [46.5, 7750]	
**easTILs (%)**			
Mean (SD)	19.2 (18.0)	23.1 (17.6)	0.304
Median [Min, Max]	14.6 [1.90, 68.4]	19.9 [1.31, 69.6]	

*Wilcoxon rank-sum test

Supplementary Table 7. Distribution (cell densities=number of positive cells/mm^2) of immune cell subpopulations (single and double markers, n=415 for total and for stromal area, n=407 for intra-tumoral area) according to the *TP53* mutational status;

	**TP53 wild-type** (n=246)	**TP53 mutated** (n=169)	**p-value***
**T helpers (CD4+)**			
**Total area**			
Mean (SD)	40.3 (151)	114 (428)	0.11
Median [Min, Max]	0 [0, 1420]	1.28 [0, 3580]	
**Intra-tumoral area**			
Mean (SD)	44.0 (244)	57.2 (244)	0.172
Median [Min, Max]	0 [0, 2860]	0 [0, 1970]	
**Stromal area**			
Mean (SD)	42.1 (176)	133 (548)	0.162
Median [Min, Max]	0 [0, 1930]	0 [0, 5460]	
**Cytotoxic T-cells (CD8+)**			
**Total area**			
Mean (SD)	96.3 (236)	185 (504)	**0.018**
Median [Min, Max]	27.2 [0, 2190]	42.9 [0, 5520]	
**Intra-tumoral area**			
Mean (SD)	44.6 (122)	93.4 (368)	**0.030**
Median [Min, Max]	7.99 [0, 1140]	15.2 [0, 4330]	
**Stromal area**			
Mean (SD)	118 (271)	221 (545)	**0.017**
Median [Min, Max]	29.4 [0, 2340]	53.4 [0, 5830]	
**Macrophages (CD68+)**			
**Total area**			
Mean (SD)	47.6 (79.6)	73.1 (160)	0.051
Median [Min, Max]	15.0 [0, 533]	27.8 [0, 1610]	
**Intra-tumoral area**			
Mean (SD)	31.6 (60.0)	35.9 (63.6)	0.13
Median [Min, Max]	4.50 [0, 376]	11.2 [0, 408]	
**Stromal area**			
Mean (SD)	65.1 (116)	108 (289)	**0.044**
Median [Min, Max]	16.1 [0, 696]	31.2 [0, 3210]	
**Regulatory T-cells (FOXP3+)**			
**Total area**			
Mean (SD)	1.18 (6.41)	3.44 (13.5)	0.187
Median [Min, Max]	0 [0, 89.5]	0 [0, 108]	
**Intra-tumoral area**			
Mean (SD)	0.366 (2.65)	1.61 (6.77)	**0.018**
Median [Min, Max]	0 [0, 27.4]	0 [0, 55.8]	
**Stromal area**			
Mean (SD)	1.97 (12.7)	4.60 (22.4)	0.197
Median [Min, Max]	0 [0, 184]	0 [0, 231]	
**PD-L1+ CD4+ T-cells**			
**Total area**			

Mean (SD)	15.0 (75.6)	68.4 (353)	**0.048**
Median [Min, Max]	0 [0, 778]	0 [0, 3220]	
**Intra-tumoral area**			
Mean (SD)	13.0 (88.9)	34.4 (182)	**0.004**
Median [Min, Max]	0 [0, 1260]	0 [0, 1840]	
**Stromal area**			
Mean (SD)	17.8 (116)	82.2 (459)	0.202
Median [Min, Max]	0 [0, 1600]	0 [0, 4810]	
**PD-L1+ CD8+ T-cells**			
**Total area**			
Mean (SD)	12.1 (65.5)	44.5 (248)	**0.002**
Median [Min, Max]	0 [0, 901]	1.17 [0, 2950]	
**Intra-tumoral area**			
Mean (SD)	5.88 (31.2)	30.6 (217)	**0.010**
Median [Min, Max]	0 [0, 419]	0 [0, 2720]	
**Stromal area**			
Mean (SD)	14.7 (70.6)	51.6 (261)	**0.006**
Median [Min, Max]	0 [0, 933]	0 [0, 3150]	
**PD-L1+ CD68+ cells**			
**Total area**			
Mean (SD)	4.76 (20.1)	19.8 (110)	**0.001**
Median [Min, Max]	0 [0, 202]	0 [0, 1320]	
**Intra-tumoral area**			
Mean (SD)	1.95 (8.70)	6.63 (26.1)	**0.005**
Median [Min, Max]	0 [0, 88.9]	0 [0, 195]	
**Stromal area**			
Mean (SD)	7.39 (36.9)	32.6 (210)	**0.001**
Median [Min, Max]	0 [0, 460]	0 [0, 2630]	
**PD-L1+ Regulatory T-cells**			
**Total area**			
Mean (SD)	0.54 (4.67)	1.95 (8.94)	**0.003**
Median [Min, Max]	0 [0, 69.6]	0 [0, 90.4]	
**Intra-tumoral area**			
Mean (SD)	0.072 (0.764)	0.935 (4.43)	**0.006**
Median [Min, Max]	0 [0, 11.0]	0 [0, 31.9]	
**Stromal area**			
Mean (SD)	1.11 (10.5)	2.71 (16.2)	**0.009**
Median [Min, Max]	0 [0, 159]	0 [0, 193]	
**PD-1+ CD4+ T-cells**			
**Total area**			
Mean (SD)	10.3 (59.3)	31.3 (121)	0.127
Median [Min, Max]	0 [0, 863]	0 [0, 883]	
**Intra-tumoral area**			
Mean (SD)	3.61 (19.4)	15.5 (99.8)	**0.028**
Median [Min, Max]	0 [0, 238]	0 [0, 1210]	
**Stromal area**			
Mean (SD)	12.9 (68.9)	38.3 (169)	0.183
Median [Min, Max]	0 [0, 969]	0 [0, 1740]	

**PD-1+ CD8+ T-cells**			
**Total area**			
Mean (SD)	9.06 (40.4)	28.8 (116)	**0.041**
Median [Min, Max]	0 [0, 424]	0 [0, 945]	
**Intra-tumoral area**			
Mean (SD)	6.18 (38.1)	24.7 (219)	0.086
Median [Min, Max]	0 [0, 475]	0 [0, 2770]	
**Stromal area**			
Mean (SD)	10.4 (46.8)	32.0 (114)	**0.018**
Median [Min, Max]	0 [0, 521]	0 [0, 945]	
**PD-1+ CD68+ macrophages**			
**Total area**			
Mean (SD)	1.26 (6.64)	6.12 (39.2)	**0.014**
Median [Min, Max]	0 [0, 83.7]	0 [0, 468]	
**Intra-tumoral area**			
Mean (SD)	0.156 (0.981)	1.95 (10.2)	**0.001**
Median [Min, Max]	0 [0, 9.17]	0 [0, 111]	
**Stromal area**			
Mean (SD)	1.79 (8.87)	11.6 (84.9)	0.065
Median [Min, Max]	0 [0, 96.3]	0 [0, 950]	
**PD-1+ Regulatory T-cells**			
**Total area**			
Mean (SD)	0.307 (1.81)	1.15 (6.28)	0.164
Median [Min, Max]	0 [0, 23.2]	0 [0, 62.8]	
**Intra-tumoral area**			
Mean (SD)	0.139 (1.05)	0.455 (3.91)	0.513
Median [Min, Max]	0 [0, 11.0]	0 [0, 47.8]	
**Stromal area**			
Mean (SD)	0.477 (3.14)	1.65 (10.1)	0.186
Median [Min, Max]	0 [0, 41.9]	0 [0, 92.4]	

*Wilcoxon rank-sum test

Supplementary Table 8. Distribution of the normalized mixing score (median value) spatial metric of cancer-immune cells interaction (derived from the multiplex fluorescence analysis) according to the *TP53* mutational status in the whole population

Radius	Cytotoxic T-cells		Immune cells*		T-helpers		T-regulatory cells		Macrophages	
	TP53-mutated	TP53-wild type	TP53-mutated	TP53-wild type	TP53-mutated	TP53-wild type	TP53-mutated	TP53-wild type	TP53-mutated	TP53-wild type
**50**	0.15	0.10	0.27	0.27	0.04	0.01	0.13	0.00	0.23	0.23
**100**	0.28	0.23	0.45	0.48	0.11	0.04	0.34	0.06	0.56	0.46
**150**	0.41	0.34	0.60	0.62	0.15	0.12	0.49	0.21	0.66	0.61
**200**	0.50	0.45	0.69	0.70	0.23	0.22	0.56	0.29	0.68	0.66
**250**	0.56	0.54	0.74	0.75	0.32	0.31	0.58	0.36	0.75	0.75
**300**	0.61	0.57	0.78	0.79	0.41	0.40	0.63	0.46	0.79	0.80
**350**	0.67	0.65	0.83	0.84	0.45	0.43	0.73	0.52	0.83	0.85
**400**	0.71	0.68	0.88	0.88	0.49	0.48	0.74	0.57	0.85	0.90
**450**	0.73	0.77	0.89	0.91	0.51	0.52	0.77	0.59	0.86	0.93
**500**	0.75	0.81	0.91	0.93	0.53	0.54	0.79	0.63	0.88	0.96
**550**	0.76	0.81	0.92	0.94	0.54	0.56	0.79	0.63	0.90	0.97
**600**	0.77	0.82	0.92	0.93	0.55	0.55	0.80	0.64	0.91	0.97

*Immune cells include cytotoxic T-cells, T-helpers, T-regulatory cells, macrophages and the respective co-expression of PD-L1/PD-1 checkpoints in each of these cell subsets

Supplementary Table 9. REMARK checklist<br>Item to be reported		Page no.
**INTRODUCTION**		
1	State the marker examined, the study objectives, and any pre-specified hypotheses.	4
**MATERIALS AND METHODS**		
Patients		
2	Describe the characteristics (e.g., disease stage or co-morbidities) of the study patients, including their source and inclusion and exclusion criteria.	15, Figure 1
3	Describe treatments received and how chosen (e.g., randomized or rule-based).	15
Specimen characteristics		
4	Describe type of biological material used (including control samples) and methods of preservation and storage.	15-18
Assay methods		
5	Specify the assay method used and provide (or reference) a detailed protocol, including specific reagents or kits used, quality control procedures, reproducibility assessments, quantitation methods, and scoring and reporting protocols. Specify whether and how assays were performed blinded to the study endpoint.	15-21, Suppl. Table 11, Suppl. Figure 7
Study design		
6	State the method of case selection, including whether prospective or retrospective and whether stratification or matching (e.g., by stage of disease or age) was used. Specify the time period from which cases were taken, the end of the follow-up period, and the median follow-up time.	15,21, Fig. 1
7	Precisely define all clinical endpoints examined.	22
8	List all candidate variables initially examined or considered for inclusion in models.	22
9	Give rationale for sample size; if the study was designed to detect a specified effect size, give the target power and effect size.	NA
Statistical analysis methods		
10	Specify all statistical methods, including details of any variable selection procedures and other model-building issues, how model assumptions were verified, and how missing data were handled.	21-22
11	Clarify how marker values were handled in the analyses; if relevant, describe methods used for cutpoint determination.	16-17, 21 -22
**RESULTS**		
Data		
12	Describe the flow of patients through the study, including the number of patients included in each stage of the analysis (a diagram may be helpful) and reasons for dropout. Specifically, both overall and for each subgroup extensively examined report the numbers of patients and the number of events.	5, Fig. 1, Suppl. Table 1
13	Report distributions of basic demographic characteristics (at least age and sex), standard (disease-specific) prognostic variables, and tumor marker, including numbers of missing values.	Suppl. Table 1
Analysis and presentation		
14	Show the relation of the marker to standard prognostic variables.	Fig. 2, Suppl. Table 2-3
15	Present univariable analyses showing the relation between the marker and outcome, with the estimated effect (e.g., hazard ratio and survival probability). Preferably provide similar analyses for all other variables being analyzed. For the effect of a tumor marker on a time-to-event outcome, a Kaplan-Meier plot is recommended.	Fig. 2 & 3, Suppl. Fig. 1 & 3
16	For key multivariable analyses, report estimated effects (e.g., hazard ratio) with confidence intervals for the marker and, at least for the final model, all other variables in the model.	Fig. 2 & 3, Suppl. Fig. 1 & 3
17	Among reported results, provide estimated effects with confidence intervals from an analysis in which the marker and standard prognostic variables are included, regardless of their statistical significance.	Fig. 2 & 3, Suppl. Fig. 1 & 3

18	If done, report results of further investigations, such as checking assumptions, sensitivity analyses, and internal validation.	NA
**DISCUSSION**		
19	Interpret the results in the context of the pre-specified hypotheses and other relevant studies; include a discussion of limitations of the study.	10-14
20	Discuss implications for future research and clinical value.	10-14

Supplementary Table 10. List of all participating centers in the clinical study

Country	Site	Comment	EC	Address	EC reference numbers
**Belgium**	101		Comité d'Ethique Institut Jules Bordet	Comité d'Ethique Institut Jules Bordet<br>Bld. de Waterloo 121<br>1000 Brussels<br>Belgium	DB/CG/1026
**Belgium**	155		Comité d'Ethique Hospitalo-Facultaire Universitaire de Liège	Comité d'Ethique Hospitalo-Facultaire Universitaire de Liège<br>Centre Hospitalier Universitaire du Sart Tilman, B35<br>4000 Sart Tilman par Liège 1<br>Belgium	2004/72
**Belgium**	1234		Ethische Comité Sint Augustinus	Ethische Comité Sint Augustinus<br>Oosterveldlaan 24<br>2610 Wilrijk<br>Belgium	10994
**Switzerland**	935	Not EORTC responsibility --> through SAKK	Kantonale Ethikkomission AG/S0	Kantonale Ethikkomission AG/S0<br>Bachstr. 15<br>5001 Aarau	
**Switzerland**	451	Not EORTC responsibility --> through SAKK	Commission Cantonale d'Ethique de la Recherche	Commission Cantonale d'Ethique de la Recherche<br>Rue Adrien-Lachenal 8<br>1207 Geneva<br>Switzerland<br>Phone +41 225465028<br>Fax +41 22 372 90 20<br>Email ccer@etat.ge.ch – Central EC	10994
**Switzerland**	453	Not EORTC responsibility --> through SAKK	Kantonale Ethikkommission Zürich	Info.KEK@kek.zh.ch	10994
**Switzerland**	454	Not EORTC responsibility --> through SAKK	Kantonale Ethikkommission Bern (KEK)	Kantonale Ethikkommission Bern (KEK)<br>Murtenstrasse 31<br>3010 Bern<br>Switzerland<br>Phone +41 316337070<br>Fax +41 316337071<br>Email info.kek.kapa@gef.be.ch	10994
**France**	1 EC For all sites	Central submission to 1 EC	Comité de protection des personnes "Sud-Ouest et Outre-mer III", Hôpital Pellegrin	Comité de protection des personnes "Sud-Ouest et Outre-mer III", Hôpital Pellegrin<br>Bâtiment 1A - Service de pharmacologie clinique<br>Place Amélie-Raba-Léon<br>33076 Bordeaux<br>France	10994

**UK**	1 EC For all sites	South East Scotland Research Ethics Service	Not EORTC responsibility --> through NHS National Services Scotland (ACCOG)	South East Scotland Research Ethics Service<br>Waverley Gate<br>2-4 Waterloo Place<br>Edinburgh<br>EH1 3EG	10994
**The Netherlands**	1 EC For all sites	310 act as Central EC	Commissie Medische Ethiek Leiden Universitair Medisch Centrum	Commissie Medische Ethiek Leiden Universitair Medisch Centrum<br>Albinusdreef, 2.<br>Postbus 9600<br>2300 RC Leiden<br>The Netherlands	P01.043
**Poland**	550		Komisja Bioetyczna przy Centrum Onkologii Instytucie	Komisja Bioetyczna przy Centrum Onkologii Instytucie<br>Ul. Roentgena, 5<br>02-781 Warsaw<br>POLAND	15/2002
**Poland**	551		Niezaleznej Komisji Bioetycznej Do Spraw Badan Naukowych	Niezaleznej Komisji Bioetycznej Do Spraw Badan Naukowych<br>Przy Akademli Medyczney W Gdansku<br>Ul. M. Sklodowskiej-Curie 3A<br>80 210 Gdansk<br>POLAND	819/2000
**Portugal**	1 EC For all sites	Central submission to 1 EC	CEIC - Comissão de Ética para a Investigação Clínica	CEIC - Comissão de Ética para a Investigação Clínica<br>Parque de Saúde de Lisboa<br>Avenida do Brasil, 53 - Pav 17-A 1749-004 Lisboa<br>Portugal	DVIG/UGEC
**Sweden**	1 EC For all sites	Central Ethical Review Board/ Centrala Etikprövningsnämnden (EPN)	Not EORTC responsibility --> through Karolinska (SWEBCG)	Central Ethical Review Board/ Centrala Etikprövningsnämnden (EPN)<br>c/o Vetenskapsrådet<br>101 38 Stockholm<br>Sweden	10994
**Slovenia**	969		Komisija Republike Slovenije za medicinsko etiko	Komisija Republike Slovenije za medicinsko etiko (National Medical Ethics Committee)<br>Zaloška c. 7<br>SI 1525 Ljubljana<br>Slovenia	10994

Supplementary Table 11. List of antibodies and experimental conditions used for the multiplex immunofluorescence method

<table>
<tr><th>Order</th><th>Antigen retrieval*</th><th>Marker</th><th>Clone</th><th>Host Species</th><th>Dilution</th><th>Company</th><th>Fluorophore</th></tr>
<tr><td>1</td><td>pH9</td><td>CD68</td><td>PG-M1</td><td>Mouse</td><td>1:400</td><td>Agilent</td><td>Opal 480</td></tr>
<tr><td>2</td><td>pH6</td><td>PD-1</td><td>NAT105</td><td>Mouse</td><td>1:100</td><td>Abcam</td><td>Opal 650</td></tr>
<tr><td>3</td><td>pH6</td><td>CD4</td><td>4B12</td><td>Mouse</td><td>1:100</td><td>Agilent</td><td>Opal 520</td></tr>
<tr><td>4</td><td>pH6</td><td>CD8a</td><td>C8/144B</td><td>Mouse</td><td>1:200</td><td>Thermo Fisher</td><td>Opal 570</td></tr>
<tr><td>5</td><td>pH6</td><td>PD-L1</td><td>SP142</td><td>Rabbit</td><td>1:400</td><td>Abcam</td><td>Opal 540</td></tr>
<tr><td>6</td><td>pH6</td><td>FoxP3</td><td>D6O8R</td><td>Rabbit</td><td>1:300</td><td>Cell Signaling</td><td>Opal 620</td></tr>
<tr><td rowspan="3">7</td><td rowspan="3">pH6</td><td>Cytokeratin</td><td>AE1/AE3</td><td>Mouse</td><td>1:400</td><td>Dako</td><td rowspan="3">Opal 690</td></tr>
<tr><td>PanCK</td><td>C-11</td><td>Mouse</td><td>1:500</td><td>Abcam</td></tr>
<tr><td>E-cadherin</td><td>36/E</td><td>Mouse</td><td>1:2000</td><td>BD Biosciences</td></tr>
<tr><td>8</td><td>pH6</td><td>DAPI</td><td>-</td><td>-</td><td>-</td><td>Perkin Elmer</td><td>-</td></tr>
</table>

*Antigen retrieval performed in an automated Leica Bond RX^m Research Stainer at 95 ^{0}C, 20min. The ImmPRESS® HRP Anti-Mouse IgG (Peroxidase) (Cat. No: MP-7402-50), Anti-Rabbit IgG (Peroxidase) Polymer Detection Kits, made in Horse (Cat No: MP-7401-50) (Vector Laboratories) and Opal Polymer HRP (Mouse + Rabbit, ARH1001EA, Perkin Elmer) were used as secondary antibodies. 4′,6-diamidino-2-phenylindole (DAPI) was used in order the nuclei to be visualized. The ProLong^{TM} Diamond Antifade Mountant (ThermoFisher, Waltham, MA, USA) was used as a mounting medium. The concept of sequential staining (regardless of antibody species) and signal detection is based on the use of fluorophores followed by microwave treatment (for the removal of primary and secondary HRP-conjugated antibodies -once the previous target has been already detected- and for reduction of auto-fluorescence), avoiding cross-reactivity.

Supplementary Table 12. Cell classification based on the markers derived from the multiplex immunofluorescence method

Cell subclass /marker	CD4	CD8	FoxP3	CD68	PD-L1	PD-1
CD4	+		-			
CD8		+				
CD68				+		
T-regulatory cells			+			
PD-L1+CD4+	+				+	
PD-L1+CD8+		+			+	
PD-L1+CD68+				+	+	
PD-L1+ T-regulatory cells			+		+	
PD-1+CD4+	+					+
PD-1+CD8+		+				+
PD-1+CD68+				+		+
PD-1+ T-regulatory cells			+			+

Supplementary Figure 1. Forest plots on prognostic effect of digital TILs on progression-free survival (PFS); A. Univariate model; B. Multivariable model, adjusted for tumor size, nodal status, treatment and stratified by subtype

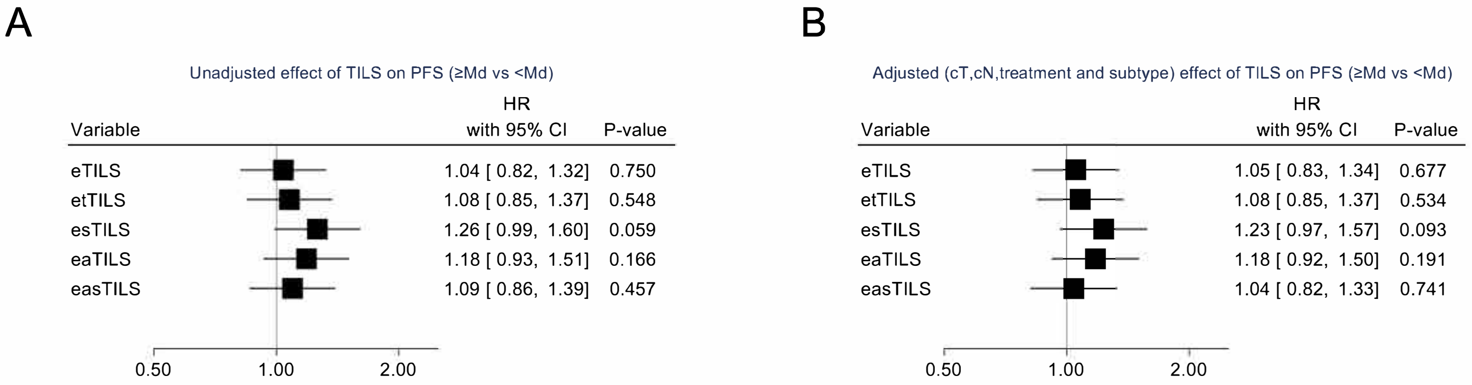

Supplementary Figure 2. Correlation matrix for digital TILs and multiplex immunofluorescence immune subpopulations (n = 387) for (A) single marker and (B) double marker expression in total area; *** p<0.001

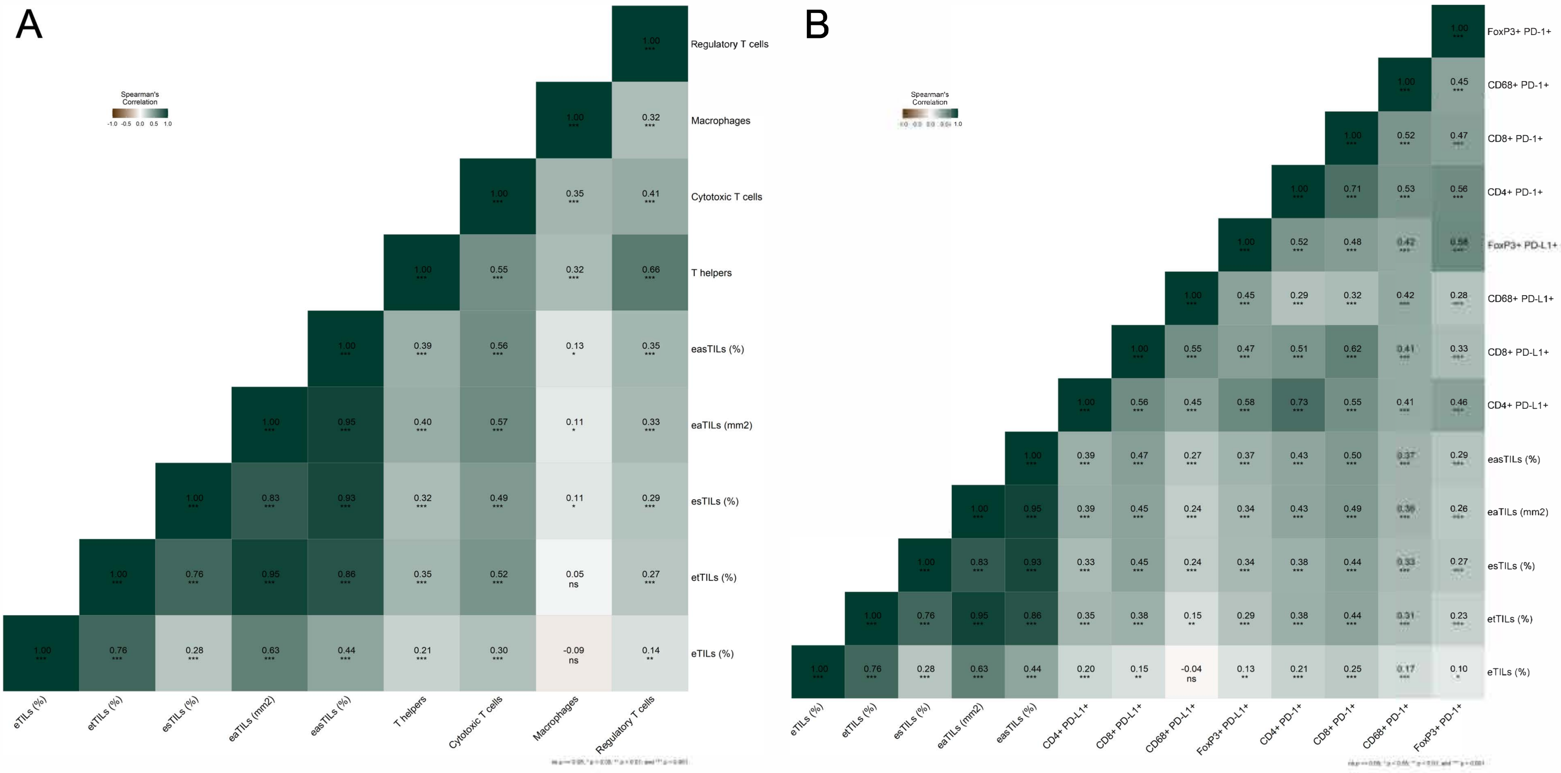

Supplementary Figure 3. Forest plots on prognostic effect of single and double multiplex markers on progression-free survival (PFS); A. Univariate model; B. Multivariable model, adjusted for tumor size, nodal status, treatment and stratified by subtype

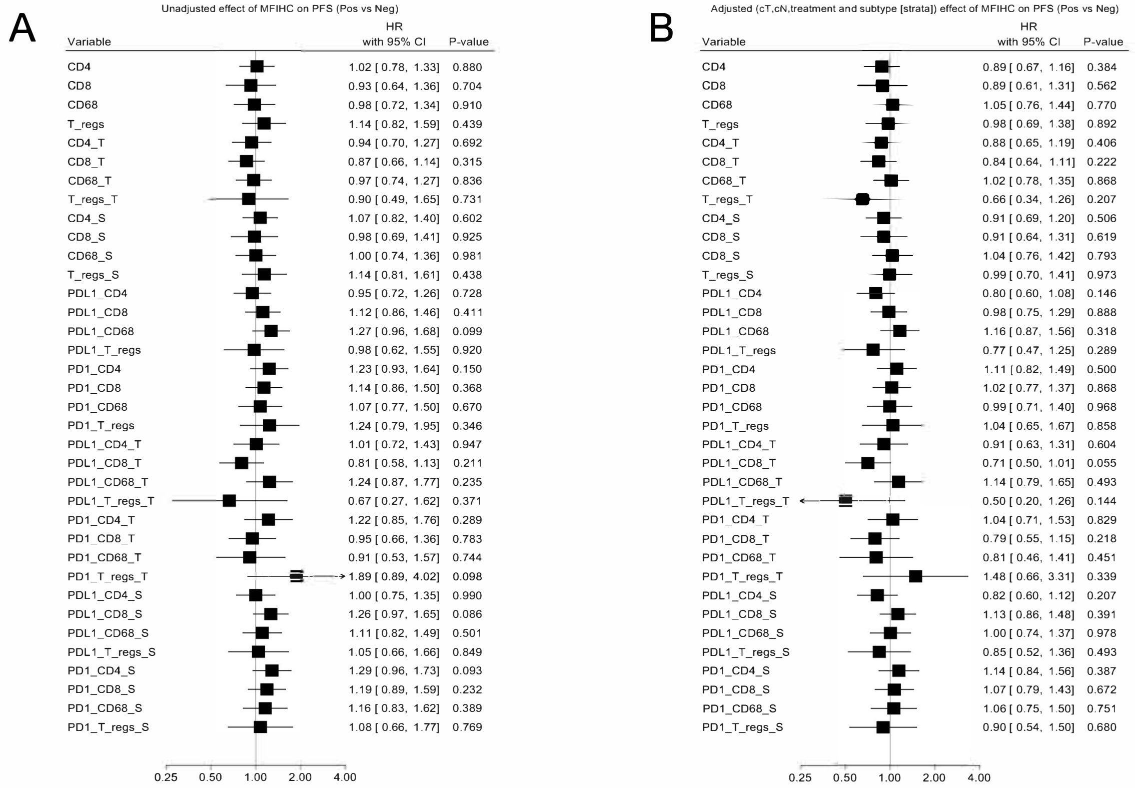

Supplementary Figure 4. Entropy gradient slopes according to pCR status in triple-negative subtype for interactions of tumor cells with all immune cells (A), cytotoxic T-cells (B), T-helpers (C), T-regulatory cells (D), macrophages (E); each curve represent the median of all radii for the specific cell interactions in an attraction- or repulsion-like pattern

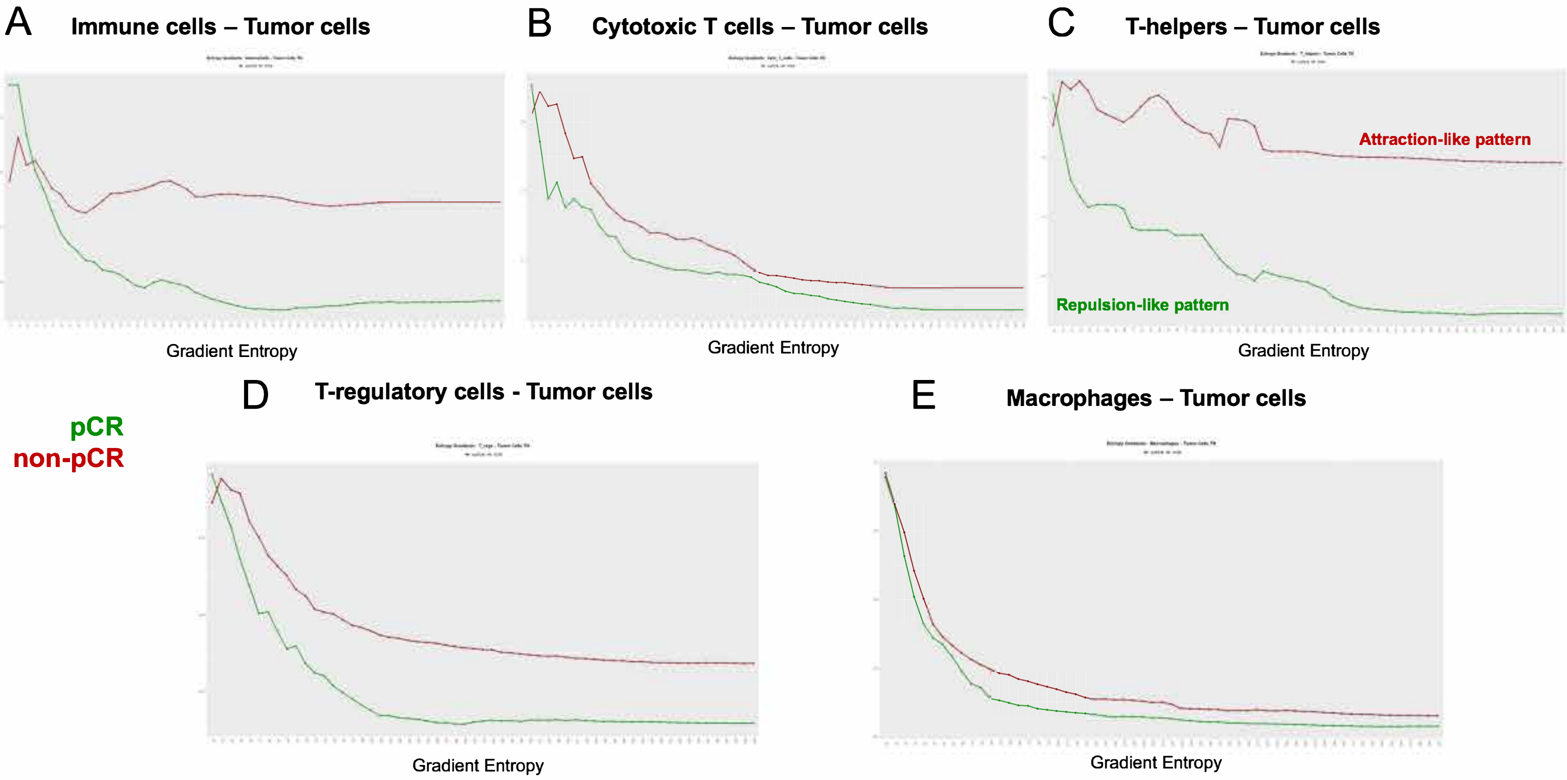

Supplementary Figure 5. Normalized mixing score according to TP53 mutational status (representative images of the spatial distributions, A) in the overall population patients for interactions of tumor cells with all immune cells (B), cytotoxic T-cells (C), T-regulatory cells (D), macrophages (E) and T-helpers (F)

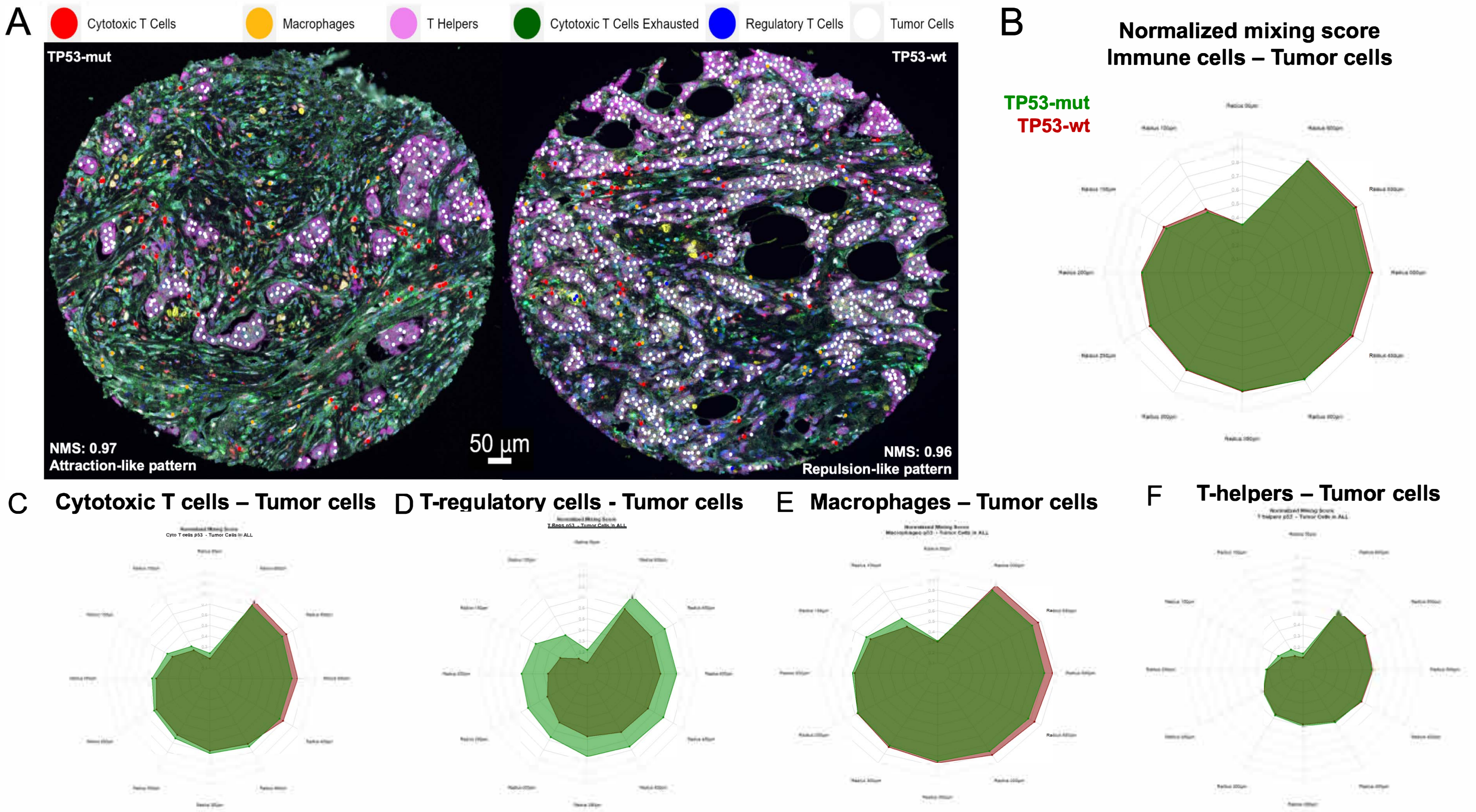

Supplementary Figure 6. Entropy gradient slopes slopes according to TP53 mutational status in the overall population patients for interactions of tumor cells with all immune cells (A), cytotoxic T-cells (B), T-helpers (C), T-regulatory cells (D), macrophages (E); each curve represent the median of all radii for the specific cell interactions in an attraction- or repulsion-like pattern

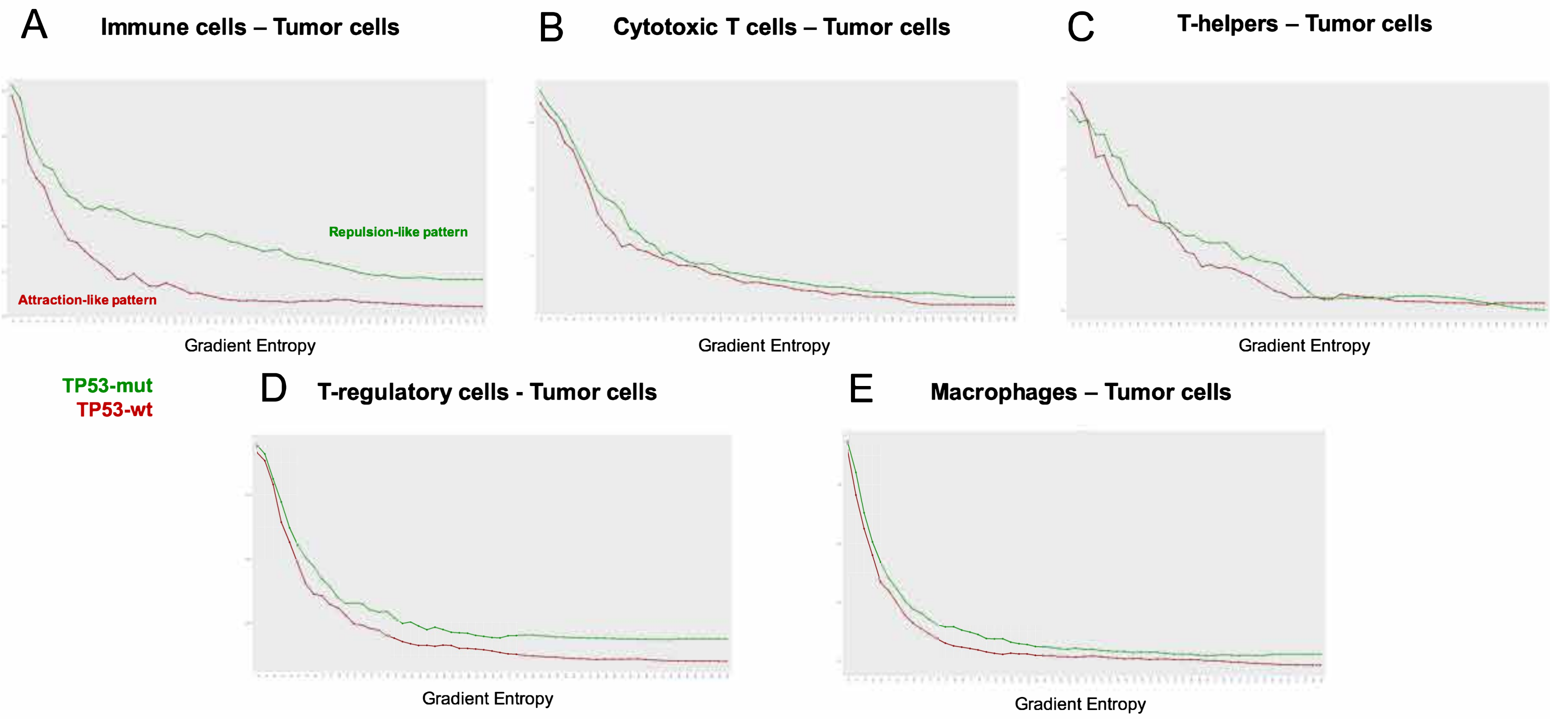

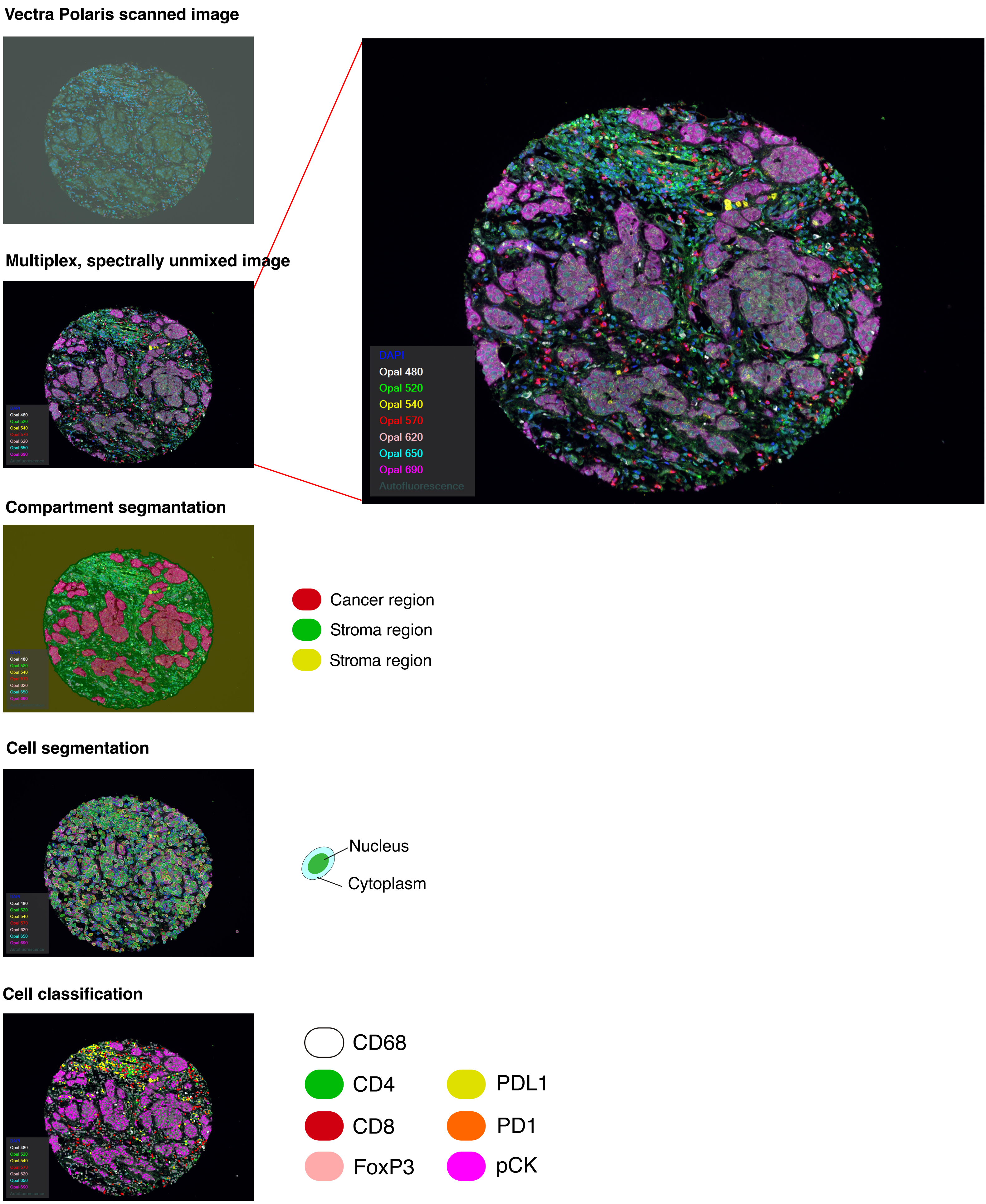

Supplementary Figure 7. Workflow for the multiplex immunofluorescence multispectral image analysis
